# Unveiling the anti-aging of radix saposhnikoviae: A metabolomic study in *Drosophila*

**DOI:** 10.1371/journal.pone.0330274

**Published:** 2025-08-20

**Authors:** Liwei Jia, Xianglin An, Yan Liu, Xin Meng

**Affiliations:** School of Pharmacy, Heilongjiang University of Chinese Medicine, Harbin, Heilongjiang, China; Baylor College of Medicine, UNITED STATES OF AMERICA

## Abstract

Aging is characterized by a progressive decline in physiological functions and an increased susceptibility to age-related diseases, yet effective interventions remain limited. Recent advancements in understanding the molecular mechanisms of aging have highlighted pathways such as insulin-like signaling, mTOR, and sirtuins. Meanwhile, traditional medicinal herbs are increasingly recognized for their potential to modulate these pathways. However, comprehensive analyses investigating how these herbs influence multiple aging-related metabolic pathways simultaneously remain scarce. This study examines the anti-aging and antioxidant effects of Radix Saposhnikoviae (*Fangfeng*) through metabolomic analysis using *Drosophila melanogaster* as a model organism. Our findings indicate that different *Fangfeng* preparations significantly extended the lifespan of *Drosophila* to varying extents. Utilizing nuclear magnetic resonance (NMR) metabolomics, we identified key metabolic pathways modulated by *Fangfeng*, including those related to energy metabolism, oxidative stress response, lipid metabolism, protein homeostasis, and inflammatory processes-each closely associated with aging. The results revealed significant regulation of these pathways, particularly those involved in oxidative stress and energy homeostasis, which are central to the aging process. These findings underscore the potential of Radix Saposhnikoviae as a promising medicinal herb for modulating key biochemical pathways associated with aging and oxidative stress. This study provides a scientific basis for the integration of traditional herbal medicine into contemporary anti-aging strategies, contributing to the expanding field of aging research.

## Introduction

Aging is a complex, multifactorial biological process characterized by the progressive decline of physiological functions, leading to increased susceptibility to various diseases, including cardiovascular disorders, neurodegenerative diseases, and metabolic syndromes [1–3]. This gradual deterioration is driven by a combination of genetic, environmental, and lifestyle factors, each contributing to the intricate network of biological pathways that regulate the aging process [4,5]. As global life expectancy continues to rise, the burden of age-related conditions on public health systems has become increasingly pronounced. Consequently, understanding the mechanisms underlying aging and developing strategies to delay or mitigate its effects have emerged as critical objectives in biomedical research. These efforts aim to extend the duration of healthy living, known as healthspan, while also reducing the societal and economic burdens of age-related diseases, ultimately enhancing the quality of life for aging populations [6].

Recent research has identified several key pathways involved in aging, including the insulin-like signaling (ILS) pathway [7], the mechanistic target of rapamycin (mTOR) pathway [8], and sirtuins [9], along with the coenzyme NAD+ [10]. These pathways have been widely studied for their roles in regulating aging and related diseases. The ILS pathway, for example, has been shown to significantly influence lifespan, as demonstrated by the discovery that mutations in the daf-2 gene in Caenorhabditis elegans can nearly double the organism’s lifespan [11]. This finding highlights the profound impact that alterations in growth and metabolic signaling can have on longevity. Similarly, inhibition of the mTOR pathway, initially studied for its role in immune modulation, has been validated across various model organisms, including yeast, worms, flies, and mice, as a means to delay aging and extend lifespan. The role of mTOR in aging is particularly compelling due to its central position in cellular metabolism, nutrient sensing, and autophagy, processes that are critical in maintaining cellular homeostasis during aging [8,12].

Sirtuins, particularly the Sir2 gene in yeast, have also garnered attention for their ability to extend lifespan through epigenetic regulation. Sirtuins are a family of NAD + -dependent deacetylases that influence aging by modulating chromatin structure, DNA repair, and metabolic regulation [9,13]. These findings have been echoed in other organisms, including mammals, underscoring their potential in extending healthspan. NAD + , a coenzyme involved in redox reactions and a substrate for sirtuins, has also been a focus of aging research. NAD+ levels decline with age, and restoring these levels through supplementation has been shown to improve metabolic function, reduce oxidative stress, and extend lifespan in animal models [14].

Despite these advancements, the translation of these findings into effective clinical interventions remains a significant challenge. Pharmacological agents such as rapamycin, metformin and NAD+ precursors [15] have shown promise in preclinical studies and are currently under investigation in clinical trials. Rapamycin, a mTOR inhibitor, has demonstrated lifespan extension in various model organisms and is being explored for its potential to delay age-related diseases in humans [16]. Metformin, a drug commonly used to treat type 2 diabetes, has also been studied for its anti-aging effects due to its ability to modulate metabolic pathways, reduce oxidative stress, and activate AMPK, a key energy sensor in cells [17] NAD+ precursors, such as nicotinamide riboside (NR) and nicotinamide mononucleotide (NMN), are being investigated for their potential to boost NAD+ levels and improve mitochondrial function, thereby delaying the onset of age-related diseases.

However, the need for novel approaches and complementary therapies remains critical, particularly those that integrate traditional medicinal practices with modern scientific methodologies. Among the complementary therapies, traditional herbal medicine has garnered considerable attention for its potential in anti-aging research. Plant-derived compounds, due to their wide range of bioactive constituents, have been shown to interact with multiple biological pathways associated with aging. These include pathways related to oxidative stress, inflammation, and cellular senescence-key contributors to the aging process [18].

The use of herbal medicines in anti-aging is deeply rooted in traditional practices across various cultures, and modern research is increasingly validating these applications through scientific investigations. For example, Ginkgo biloba, a herb long used in traditional Chinese medicine (TCM), has been extensively studied for its neuroprotective and antioxidant properties [19]. Research has shown that Ginkgo biloba extracts can improve cognitive function, reduce oxidative damage, and enhance blood flow to the brain, making it a promising candidate for preventing age-related neurodegenerative diseases [20,21]. Similarly, Panax ginseng, another cornerstone of TCM, has been shown to modulate the immune system, reduce inflammation, and improve physical stamina, contributing to its reputation as an adaptogen that promotes longevity [22]. Curcuma longa (turmeric), widely used in Ayurvedic medicine, contains curcumin, a compound with potent anti-inflammatory and antioxidant effects. Curcumin has been shown to inhibit the activation of NF-κB, a key regulator of inflammation, and to protect against cellular damage caused by free radicals, thus offering potential benefits in delaying the aging process [23].

In the context of these broader research efforts, *Fangfeng* stands out as a well-known herb in TCM, recognized for its extensive pharmacological activities, including anti-inflammatory, immunomodulatory, antiviral, analgesic, and neuroprotective effects [24–27]. Radix Saposhnikoviae, the dried root of *Saposhnikovia divaricata* (Turcz.) Schischk., is the medicinal part traditionally used. Despite its widespread use, the pharmacological effects of *Fangfeng* remain underexplored, particularly in the context of aging and age-related diseases.

To address these gaps, this study employs advanced metabolomic analysis to systematically investigate the anti-aging effects of *Fangfeng* in a *Drosophila melanogaster* model. This model organism is widely used in aging research due to its short lifespan, well-characterized genetics, and conservation of key aging-related pathways with humans. Phytochemical analyses have shown that *Fangfeng* is rich in polysaccharides [28], coumarins [29], flavonoids [30], and volatile oils [31], with demonstrated immunomodulatory and antioxidant properties [32]. These findings suggest that the herb’s multi-component nature may influence aging-related pathways.

By comparing the antioxidant and anti-aging properties of various *Fangfeng* preparation methods (raw, stir-fried, and charred), this research aims to uncover alterations in key metabolic pathways using nuclear magnetic resonance (NMR) metabolomics. This approach not only provides a deeper understanding of the herb’s bioactivity but also contributes to the development of standardized preparation methods, ensuring consistency and reliability in clinical applications.

Previous studies by our group demonstrated that stir-frying and charring significantly altered the volatile composition of Radix Saposhnikoviae, notably increasing the content of β-bisabolene, a key anti-inflammatory compound. These processing-induced phytochemical changes may contribute to the differential bioactivities observed in this study.

Furthermore, *Fangfeng*’s antioxidant properties are of particular interest in aging research. Oxidative stress, caused by the accumulation of reactive oxygen species (ROS), is a major contributor to cellular aging and the development of age-related diseases. The ability of *Fangfeng* to scavenge free radicals and reduce oxidative damage to cells and tissues may provide a protective effect against the detrimental consequences of aging [33]. This antioxidative capacity, combined with its anti-inflammatory effects, positions *Fangfeng* as a potential candidate for developing new anti-aging therapies that target the underlying mechanisms of aging.

While modern medicine has made significant strides in understanding and potentially mitigating the effects of aging, the integration of traditional medicinal practices, such as the use of *Fangfeng* and other herbal medicines, offers a promising avenue for developing holistic and multi-targeted approaches to extending healthspan. This study, by elucidating the anti-aging properties of *Fangfeng* through advanced metabolomic techniques, contributes to this growing body of knowledge and highlights the importance of preserving and modernizing traditional herbal practices in the pursuit of longevity.

## Experimental Materials and methods

### Herbal samples

In this study, we used three different preparations of *Fangfeng*: FS, which refers to raw Fangfeng; FC, which refers to stir-fried *Fangfeng*; and FT, which refers to charred *Fangfeng*. Each preparation was subjected to a different processing method, which may influence its bioactivity.

*Fangfeng* was purchased from Sankeshu Herbal Market in Harbin, Heilongjiang Province, and identified as genuine *Fangfeng* by Dr. Wang Zhenyue.

Raw *Fangfeng* (FS): The raw herb was cleaned, moistened, sliced, and dried for further use.

Stir-fried *Fangfeng* (FC): Raw *Fangfeng* slices were stir-fried at 100°C for 4 mins, until slightly charred. The cooled slices were stored in airtight bags.

Charred *Fangfeng* (FT): Raw *Fangfeng* slices were stir-fried at 180°C for 6 mins until partially carbonized. The cooled slices were stored in airtight bags.

*Fangfeng* aqueous extract:50 g of raw, stir-fried, and charred *Fangfeng* powders were each boiled with 15 volumes of distilled water. After filtering, the residue was reboiled with 10 volumes of water. The combined filtrates were concentrated to a final volume of 50 mL, yielding a 1 g/mL aqueous extract for each preparation.

*Fangfeng* volatile oil: Samples of raw *Fangfeng*, stir-fried *Fangfeng*, and *charred Fangfeng* were ground and sieved to obtain fine powders. 50 g of each powdered sample was weighed and extracted using petroleum ether (boiling range 60°C-90°C) in a Soxhlet apparatus. The extraction process was conducted for 6h in a water bath maintained at 80°C. After extraction, the solvent was removed by rotary evaporation to recover the petroleum ether. The resulting crude extract was treated with anhydrous sodium sulfate (Na_2_SO_4_) and left to stand overnight. The supernatant was collected to obtain the volatile oils from each sample.

### Experimental animals

The black strain *Drosophila melanogaster* used in this study were obtained from the Center for Excellence in Molecular Cell Science, Chinese Academy of Sciences. The flies were maintained in an artificial climate chamber under controlled conditions: 25°C temperature, 60% humidity, and a 12h light/dark cycle.

### Medium

The basic medium was prepared by mixing glutinous corn flour, sucrose, agar powder, yeast powder, propionic acid, and distilled water. The mixture was heated, cooled to 70°C, and distributed into sterilized *Drosophila* tubes before solidifying at 4°C. For the medicated medium, *Fangfeng* aqueous extracts were added to the basic medium at low (0.02 g/mL), medium (0.04 g/mL), and high (0.08 g/mL) dosages.

Additional experimental procedures and detailed descriptions of the methodologies are provided in the supplementary materials.

#### Lifespan assay of *Drosophila melanogaster.*

**Model selection and grouping**: *Drosophila melanogaster* (black strain) were selected as the model organisms for natural aging studies. Both male and female flies were used to assess the anti-aging effects of raw *Fangfeng*, stir-fried *Fangfeng*, and charred *Fangfeng* extracts at low, medium, and high doses. The experiment aimed to observe the lifespan, reproductive capacity, climbing ability, and body weight of flies in both control and treatment groups, providing a comprehensive evaluation of the anti-aging effects of different *Fangfeng* preparations and the differences between them.

**Lifespan study**: Adult *Drosophila*, within 3d of eclosion and unmated, were collected and anesthetized using CO₂. The flies were sexed and randomly divided into ten groups: a control group and nine treatment groups (three types of *Fangfeng* extracts at three doses each). Each group consisted of four vials per sex, each containing 25 flies. The flies were fed with either basic or medicated medium, which was replaced every 5d. Mortality was recorded daily until all flies died naturally. The average lifespan, maximum lifespan, and median lifespan were calculated for each group, and survival curves were plotted. The average lifespan was defined as the mean survival time of all flies in a group, whereas the maximum lifespan was determined by averaging the survival times of the last ten individuals.

**Reproductive capacity study**: Adult flies within 8h of eclosion were collected and sexed. They were randomly divided into ten groups, similar to the lifespan study, with each group consisting of two vials per sex and 15 flies per vial. The medium was replaced every 5d. After 5d of feeding, ten male and ten female flies from each group were placed together in a vial with the corresponding medium. The number of pupae was recorded on the tenth day, and the experiment was repeated three times.

**Climbing ability study**: Adult flies within 8h of eclosion were collected and sexed. They were randomly divided into ten groups, with each group consisting of two vials per sex and 15 flies per vial. The medium was replaced every 5d. After 15d of feeding,10 male and 10 female flies from each group were placed in a collection tube. The flies were lightly tapped to the bottom of the tube, and the number of flies climbing above the 8 cm mark within one minute was recorded. This process was repeated three times to calculate the climbing index.


$$\text{Climbing index}=\frac{\text{Number of flies climbing above the 8 cm line}}{\text{Total number of flies}}\times 100\%$$

**Body Weight Analysis**: Adult *Drosophila melanogaster* were collected within 8 hours of eclosion, sexed to separate males and females, and randomly assigned to 10 groups: one control group and nine treatment groups, each receiving low, medium, or high doses of one of three Fangfeng preparations (raw, stir-fried, and charred). Each group was housed in two vials per sex, with 15 flies per vial. The flies were fed for 15 days, and after the feeding period, 10 male and 10 female flies were randomly selected from each group and placed in Eppendorf (EP) tubes. The total weight of the 10 flies from each group was measured using an electronic balance, and this procedure was repeated three times to ensure accuracy and reproducibility randomly.

#### NMR metabolomics analysis protocol.

NMR metabolomics, a powerful metabolic analysis technique, provides a unique perspective for revealing changes in metabolic networks within organisms through comprehensive and high-resolution metabolite profiles. Due to its high sensitivity, high resolution, and full-spectrum coverage, it is widely used in the life sciences. Utilizing NMR metabolomics as a tool, this study aims to comprehensively observe the metabolic changes in *Drosophila melanogaster* fed with high concentrations of various *Fangfeng* preparations, thereby gaining a more detailed understanding of the metabolic regulation mechanisms of raw and processed *Fangfeng* in the anti-aging process.

#### Grouping and administration.

Female adult *Drosophila melanogaster*, within 3d of eclosion, were collected and randomly divided into five groups, each containing 100 flies:

Control group at 3d old (K-3d), fed with a blank medium.

Control group at 30d old (K-30d), fed with a blank medium.

0.08 g/mL raw *Fangfeng* group (FS-0.08), fed with 0.08 g/mL medicated medium for 30d.

0.08 g/mL stir-fried *Fangfeng* group (FC-0.08), fed with 0.08 g/mL medicated medium for 30d.

0.08 g/mL charred *Fangfeng* group (FT-0.08), fed with 0.08 g/mL medicated medium for 30d.

All groups were cultured under identical conditions.

**Collection of *Drosophila* samples:** On the 3rd and 30th days of cultivation, the flies were subjected to a 2h starvation treatment. The flies from each group were then collected in Eppendorf (EP) tubes for metabolomic analysis, flash-frozen in liquid nitrogen, and stored at −80°C until further analysis.

**Sample preparation:** 100 mg of fly tissue was weighed into EP tubes. Then, 1.0 mL of a chloroform: methanol (2:7:1) solution was added. The mixture was homogenized in an ice-water bath, transferred to an EP tube, and vortexed for 15 mins. The homogenate was centrifuged at 13000 rpm for 15 mins at 4°C. The supernatant was transferred to a new EP tube and evaporated to dryness. The residue was dissolved in 600 μL of phosphate buffer solution containing TSP (including D_2_O, 0.05% TSP) and vortexed to dissolve. The solution was centrifuged again at 13000 rpm for 15 mins at 4°C. The supernatant was transferred to a 5 mm NMR tube for analysis [34].

¹H-NMR spectra were recorded on a Bruker AVANCE NEO 600 MHz spectrometer at 298 K using a standard one-dimensional NOESY pulse sequence with water suppression (noesypr1d). The acquisition parameters were as follows: 64 scans, 64K data points, a relaxation delay (RD) of 2.0 s, and a mixing time of 100 ms. Presaturation was applied during both the relaxation delay and mixing time to suppress the water signal.

Processing of 1H-NMR Spectra: All 1H-NMR spectra were processed using TopSpin 4.1.3 (Bruker, Germany). Fourier transformation, phase correction, and baseline adjustment were performed. Chemical shifts were calibrated using TSP (δ 0.00). The spectral region of δ 4.60–5.00 was excluded to eliminate the water peak. The spectral region of δ 0.60–10.00 was segmented and integrated. All integration data were normalized and converted into Excel format. The processed NMR data were imported into SIMCA-P14.1 (Umetrics, Sweden) software for multivariate statistical analysis.

### Measurement of antioxidant capacity

#### Measurement of DPPH radical scavenging capacity.

1mL sample solutions (0.5 μL/mL, 1.0 μL/mL, 2.0 μL/mL, 4.0 μL/mL, and 8.0 μL/mL) was accurately pipetted into separate test tubes, followed by the addition of 1 mL of DPPH standard solution (0.3 mmol/L). The mixtures were thoroughly mixed and allowed to react in the dark for 30 mins. The absorbance was then measured at 517 nm using a visible spectrophotometer (Model KJ0512121303, Shanghai Spectrum Instrument Co., Ltd.), and the result was recorded as A1. This measurement was repeated three times for each concentration. To establish the baseline, the test solution was replaced with an equal volume of anhydrous ethanol, allowed to react in the dark for 30 mins, and the absorbance was measured as A0. Similarly, the DPPH solution was replaced with 1 mL of anhydrous ethanol, reacted in the dark for 30 mins, and the absorbance was recorded as A2. The DPPH scavenging rate was calculated using the following formula:


$$\text{DPPH scavenging rate }(\%)=\left(1-\frac{A_{1}-A_{2}}{A_{0}}\right)\times 100\%\mathrm{\backslash ]}$$

#### Measurement of hydroxyl radical scavenging capacity.

Each concentration of a sample solutions (0.5 μL/mL, 1.0 μL/mL, 2.0 μL/mL, 4.0 μL/mL, and 8.0 μL/mL) was accurately pipetted (1 mL) into separate test tubes, followed by the addition of 1 mL of salicylic acid solution (1.8 mmol/L) and 1 mL of ferrous sulfate solution (1.8 mmol/L). Finally, 1 mL of 0.3% hydrogen peroxide solution was added, and the mixture was thoroughly mixed. The reaction was allowed to proceed in a 37°C water bath for 30 mins. The absorbance was measured at 510 nm using a visible spectrophotometer (Model KJ0512121303, Shanghai Spectrum Instrument Co., Ltd.), with the result recorded as A1. This measurement was repeated three times for each concentration. The test solution was replaced with an equal volume of distilled water, allowed to react in a 37°C water bath for 30 mins, and the absorbance was measured as A0. The 0.3% hydrogen peroxide solution was replaced with 1 mL of distilled water, the reaction proceeded in a 37°C water bath for 30 mins, and the absorbance was measured as A2. The hydroxyl radical scavenging rate was calculated using the following formula:


$$\text{Hydroxyl radical scavenging rate }(\%)\;=\;(\text{1}-\frac{{\text{A}}_{\text{1}}-{\text{A}}_{\text{2}}}{{\text{A}}_{\text{0}}})\times \text{ 100}\%$$

### Data analysis

**Data processing:** Experimental data were recorded and analyzed using GraphPad Prism 9 and OriginPro 2021. The results were expressed as mean ± standard deviation (Mean ± SD). Differences between groups were assessed using one-way analysis of variance (One-way ANOVA). *P < 0.05 indicates the results are statistically significant, **P < 0.01 indicates they are highly significant, ***P < 0.001 indicates they are very highly significant, and ****P < 0.0001 indicates extremely high statistical significance.

## Results and disscusion

### Influence of processing methods and dosages of *Fangfeng* on *Drosophila* lifespan

To assess whether different processing methods of Radix Saposhnikoviae influence longevity, we performed a lifespan assay using *Drosophila melanogaster* treated with raw (FS), stir-fried (FC), and charred (FT) preparations at varying concentrations.

**Fig 1:**
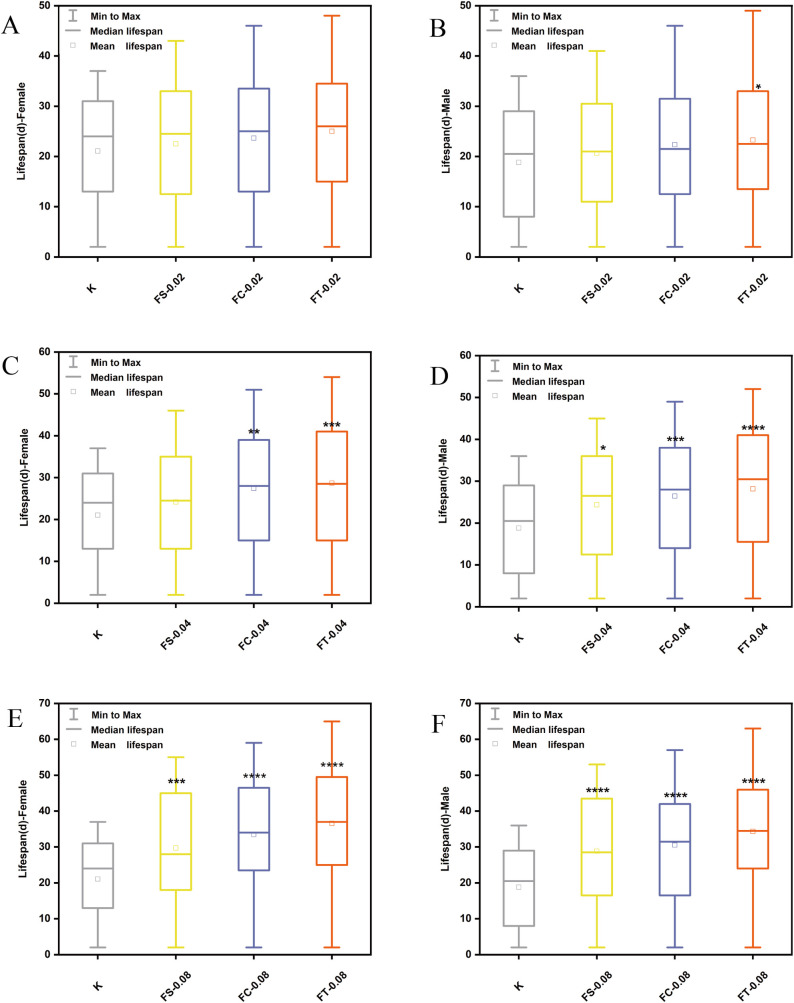
Lifespan of *Drosophila melanogaster* subjected to different processing methods of *Fangfeng* (raw *Fangfeng*, stir-fried *Fangfeng*, charred *Fangfeng*) at varying dosages. (a) Female flies at 0.02 g/mL, (b) Male flies at 0.02 g/mL, (c) Female flies at 0.04 g/mL, (d) Male flies at 0.04 g/mL, (e) Female flies at 0.08 g/mL, and (f) Male flies at 0.08 g/mL. Box plots represent minimum to maximum range, with median lifespan shown as the horizontal line and mean lifespan shown as the square within each box. Yellow represents FS-treated groups, blue represents FC-treated groups, and orange represents FT-treated groups. Statistical significance compared to the control group (K) is indicated as *P < 0.05, **P < 0.01, ***P < 0.001, and **P < 0.0001.

Fig 1 illustrates the lifespan of male and female *Drosophila melanogaster* subjected to different processing methods of *Fangfeng* (raw Fangfeng, stir-fried Fangfeng, charred Fangfeng) at varying dosages (0.02 g/mL, 0.04 g/mL, 0.08 g/mL). The results demonstrate that different processing methods significantly impact the lifespan of *Drosophila melanogaster*, with FT showing the most substantial lifespan extension across all dosages. The data reveal that, except for female flies at the 0.02 g/mL dosage, FT-treated groups exhibit significantly higher average and median lifespans compared to the control group (K) and other treated groups (FS, FC) across all dosages. At the highest dosage (0.08 g/mL), the median lifespan of female flies in the FT-treated group is approximately 37d, compared to 24d in the control group, with a maximum lifespan of 49d for the FT group versus 31d for the control group. Similarly, for male flies, the median lifespan in the FT-treated group is approximately 33d compared to 21d in the control group, with a maximum lifespan of 45d in the FT group versus 29d in the control group.

These findings underscore the clear advantage of FT treatment in significantly prolonging the lifespan of *Drosophila melanogaster*. As the dosage increased from 0.02 g/mL to 0.08g/mL, the lifespan of *Drosophila* in the FS, FC, and FT treatment groups exhibited a clear dose-dependent extension. The data demonstrate that the average and median lifespans in the FT treatment group significantly increased with higher dosages. For example, the median lifespan of female flies in the FT treatment group increased from approximately 25d at a dosage of 0.02 g/mL to 37d at a dosage of 0.08/mL This trend highlights the effectiveness of higher dosages in extending lifespan and emphasizes the dose-dependent nature of the treatments.

Kim reported that supplementing diet with 1.2 μg/mL of red ginseng increased the mean lifespan of *Drosophila* from 31.8days in the control group to 36.4 days [35]. In the present study, Fangfeng extended the mean lifespan of *Drosophila* to 37d. This outcome is comparable to the lifespan extension observed with red ginseng treatment, indicating that Fangfeng may exert a similarly robust effect on longevity. Although the difference in lifespan extension between the two treatments is marginal, it is noteworthy that Fangfeng slightly surpassed red ginseng in prolonging lifespan under our experimental conditions.

In conclusion, the results demonstrate that higher dosages of Fangfeng, particularly when processed through charred (FT), significantly extend the lifespan of *Drosophila melanogaster*. This underscores the importance of optimizing both dosage and processing methods to maximize the longevity benefits.

**Fig 2:**
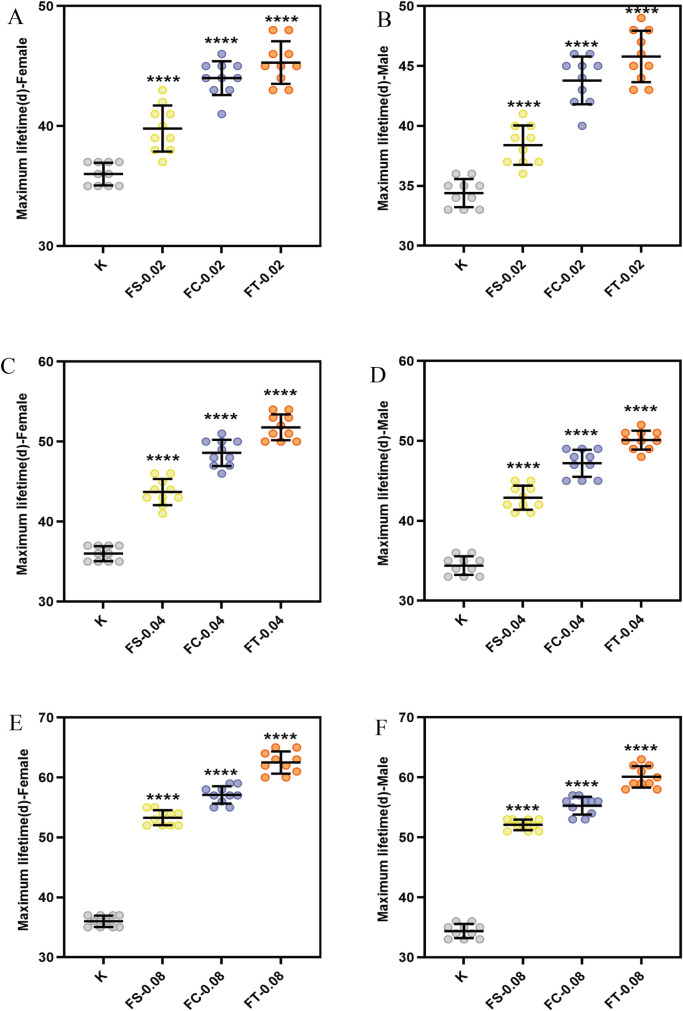
Influence of various *Fangfeng* preparations on the maximum lifespan of naturally aging *Drosophila melanogaster.* (a) Maximum lifespan of female *Drosophila* at 0.02 g/mL dosage, (b) male *Drosophila* at 0.02 g/mL, (c) female *Drosophila* at 0.04 g/mL, (d) male *Drosophila* at 0.04 g/mL, (e) female *Drosophila* at 0.08 g/mL, and (f) male *Drosophila* at 0.08 g/mL. Data points represent individual measurements (n = 8 per group), with error bars indicating standard deviation. Yellow represents FS-treated groups, blue represents FC-treated groups, and orange represents FT-treated groups, with gray representing the control group (K). Statistical significance compared to the control group is indicated as **P < 0.0001.

To determine whether Fangfeng extracts could significantly extend the maximum lifespan of *Drosophila melanogaster*, and to identify the most effective dosage, we evaluated the longest-lived individuals in each treatment group. As shown in Fig 2, the graphs depict the maximum lifespan of male and female flies subjected to different concentrations of Fangfeng extracts (raw Fangfeng, stir-fried Fangfeng, charred Fangfeng) compared to the control group (K). The concentrations tested were 0.02 g/mL, 0.04 g/mL, and 0.08 g/mL. The maximum lifespan is plotted on the y-axis, while the different treatments are indicated on the x-axis. Each data point represents the maximum lifespan of an individual *Drosophila*, with error bars indicating the standard deviation.

The primary objective of this experiment was to determine whether Fangfeng extracts could significantly extend the maximum lifespan of *Drosophila melanogaster* and to identify the most effective concentration for such an extension. The data consistently show a significant increase in the maximum lifespan of both male and female fruit flies treated with Fangfeng extracts compared to the control group. For instance, at the 0.02 g/mL concentration, all three extracts (raw Fangfeng, stir-fried Fangfeng, charred Fangfeng) significantly increased the maximum lifespan for both genders, as indicated by the higher data points and non-overlapping error bars with the control group.

There appears to be a dose-dependent response, where higher concentrations of Fangfeng extracts result in a more pronounced increase in lifespan. At the highest concentration tested (0.08 g/mL), the maximum lifespan of both male and female flies reached its peak, suggesting an optimal dose-response relationship. While both male and female fruit flies benefited from Fangfeng treatment, the extent of lifespan extension varied slightly between genders. Females generally exhibited a more substantial increase in maximum lifespan compared to males across all concentrations, indicating possible gender-specific metabolic or physiological responses to the extracts.

The results of this study demonstrate that Fangfeng extracts have a significant and dose-dependent effect on extending the maximum lifespan of *Drosophila melanogaster*. The data provide compelling evidence for the potential of Fangfeng as a natural longevity-promoting agent.

**Fig 3:**
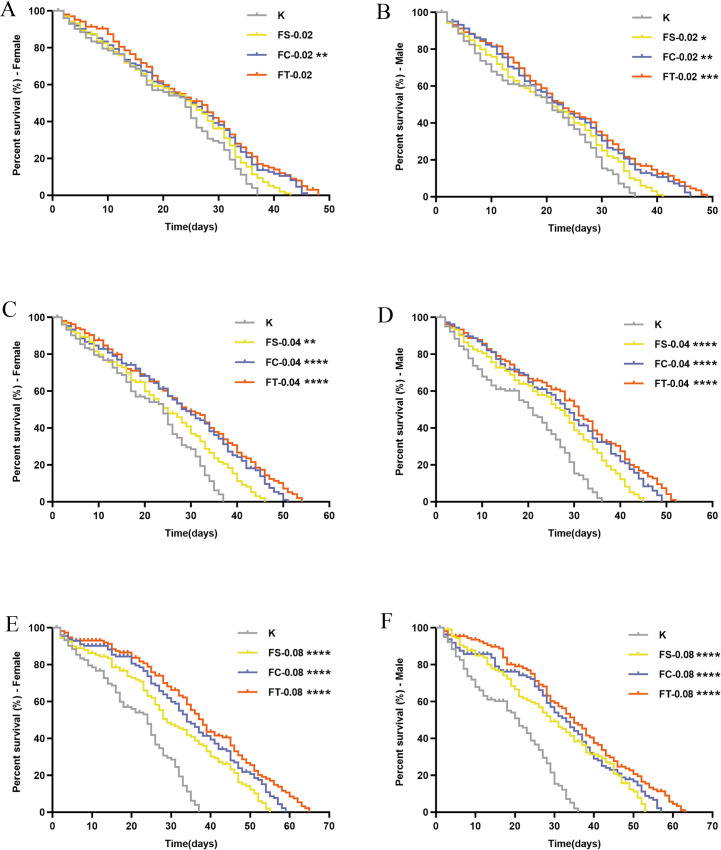
Survival curves of naturally aging *Drosophila melanogaster* treated with different processed *Fangfeng* products. (a) Survival curve of female *Drosophila* at 0.02 g/mL dosage, (b) male *Drosophila* at 0.02 g/mL, (c) female *Drosophila* at 0.04 g/mL, (d) male *Drosophila* at 0.04 g/mL, (e) female *Drosophila* at 0.08 g/mL, and (f) male *Drosophila* at 0.08 g/mL. The x-axis represents time (days), and the y-axis represents percent survival (%). Gray represents the control group (K), yellow represents FS-treated groups, blue represents FC-treated groups, and orange represents FT-treated groups. Statistical significance compared to the control group is indicated as *P < 0.05, **P < 0.01, ***P < 0.001, and **P < 0.0001.

To evaluate the impact of Fangfeng extracts on overall survival and longevity in Drosophila melanogaster, we monitored the survival rates of both male and female flies treated with different concentrations of raw, stir-fried, and charred Fangfeng. As shown in Fig 3, the survival curves illustrate the lifespan trajectories of flies under each treatment condition (raw Fangfeng, stir-fried Fangfeng, charred Fangfeng at 0.02 g/mL, 0.04 g/mL, and 0.08 g/mL) compared to the control group (K). The y-axis represents the percentage survival, while the x-axis denotes time in days. Each graph compares the survival rates across different treatment groups, illustrating the impact of Fangfeng on lifespan.

The primary objective of this experiment was to determine whether Fangfeng extracts could significantly enhance the survival rates and extend the lifespan of *Drosophila melanogaster* and to identify the most effective concentration for such an effect. The survival curves demonstrate a clear improvement in survival rates for both male and female fruit flies treated with Fangfeng extracts compared to the control group. This effect is evident across all tested concentrations (0.02 g/mL, 0.04 g/mL, 0.08 g/mL). For instance, at the 0.02 g/mL concentration, the survival curves of treated groups (raw Fangfeng, stir-fried Fangfeng, charred Fangfeng) are consistently above the control group (K), indicating a higher percentage of surviving flies over time. There appears to be a dose-dependent response to Fangfeng extracts. As the concentration increases, the survival rates of the flies also improve. At the highest concentration tested (0.08 g/mL), the survival rates are markedly higher, with the survival curves showing a significant rightward shift compared to lower concentrations and the control group. This dose-response relationship suggests that higher concentrations of Fangfeng extracts are more effective in prolonging the lifespan of *Drosophila melanogaster*. While both male and female fruit flies benefit from Fangfeng treatment, the extent of the effect varies between genders. Females generally exhibit a more pronounced increase in survival rates and lifespan extension compared to males across all concentrations. This gender-specific response might be attributed to differences in metabolic or physiological processes between male and female flies.

The results of this study indicate that Fangfeng extracts have a significant and dosedependent effect on enhancing the survival rates and extending the lifespan of *Drosophila melanogaster*. The data provide strong evidence for the potential of Fangfeng as a natural longevity-promoting agent.

### Effects of Fangfeng on the reproductive output of *Drosophila melanogaster*

**Fig 4:**
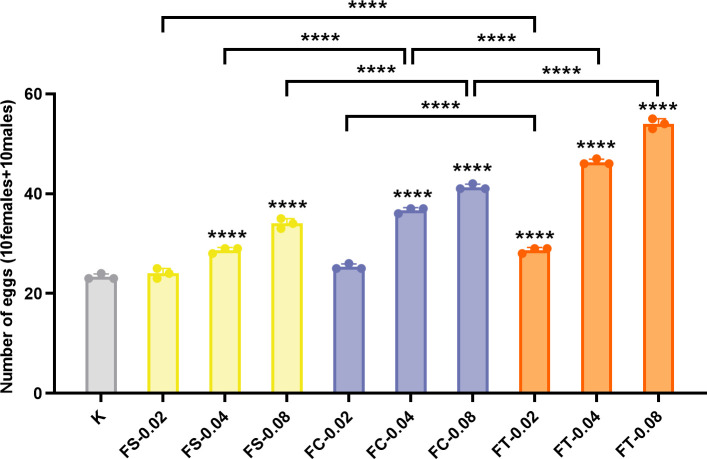
Reproductive output of *Drosophila melanogaster* treated with varying *Fangfeng* preparations at different concentrations (0.02, 0.04, and 0.08 g/mL). The x-axis represents different *Fangfeng* treatment groups (K: control, FS: raw *Fangfeng*, FC: stir-fried *Fangfeng*, FT: charred *Fangfeng*), and the y-axis represents the number of pupae produced by 10 female and 10 male *Drosophila*. Yellow columns represent FS-treated groups, blue columns represent FC-treated groups, and orange columns represent FT-treated groups. Columns show mean values ± SEM (n = 3), with individual data points representing three independent experiments. Statistical significance compared to the control group is indicated as **P < 0.0001.

To further explore whether Fangfeng affects not only longevity but also physiological functions related to fitness, we assessed the reproductive output of treated flies. Reproductive capacity is a key indicator of organismal health and vitality during aging. Fig 4 illustrates the number of pupae produced by 10 female and 10 male *Drosophila melanogaster* subjected to different processing methods of Fangfeng (raw Fangfeng, stir-fried Fangfeng, charred Fangfeng) at varying dosages (0.02 g/mL, 0.04 g/mL, 0.08 g/mL). The results demonstrate that different processing methods significantly impact the reproductive output of *Drosophila melanogaster*, with FT showing the highest pupa production across all dosages. The data reveal that FT-treated groups exhibit significantly higher pupa counts compared to the control group (K) and other treated groups (FS, FC) across all dosages.

At the highest dosage of 0.08 g/mL, the FT-treated group produced approximately 57 pupae, compared to about 25 pupae in the control group. Similarly, the FC-treated group at the 0.08 g/mL dosage produced about 50 pupae, while the FS-treated group at the same dosage produced around 42 pupae. The FT method consistently resulted in the highest pupa production, indicating a clear advantage in terms of reproductive output.

The statistical significance of these differences is highlighted by the asterisks (****) in the figure, indicating highly significant differences (p < 0.0001) between the groups. This underscores the effectiveness of higher dosages, particularly with the FT processing method, in enhancing the reproductive output of *Drosophila melanogaster*.

Overall, these findings demonstrate that higher doses of Fangfeng, particularly when processed as charred Fangfeng (FT), significantly enhance the reproductive output of *Drosophila melanogaster*. This emphasizes the importance of optimizing both dosage and processing methods to maximize reproductive benefits. However, although pupal count is a practical and widely used proxy for larval survival and potential population fitness [36], it does not directly reflect reproductive capacity. To obtain a more comprehensive assessment of reproductive health, future studies should include additional parameters such as egg hatching rates, embryonic lethality, and the proportion of unfertilized eggs.

### Effects of different preparations of *Fangfeng* on climbing ability in naturally aging *Drosophila melanogaster*

By quantitatively assessing the impact of different processing methods (raw *Fangfeng*, stir-fried *Fangfeng*, charred *Fangfeng*) and dosages (0.02 g/mL, 0.04 g/mL, 0.08 g/mL) on the climbing ability of female and male *Drosophila*, we can explore the anti-aging benefits of *Fangfeng*.

**Fig 5:**
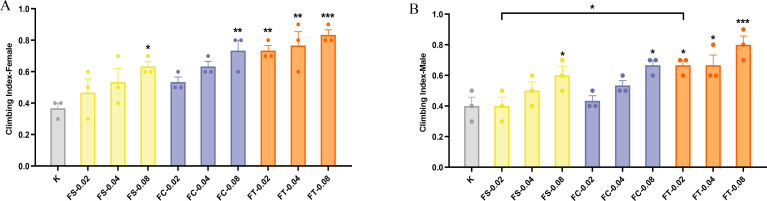
Impact of different *Fangfeng* preparations on the climbing ability of aging *Drosophila melanogaster* at 0.02, 0.04, and 0.08 g/mL dosages. The x-axis represents different *Fangfeng* treatment groups (K: control, FS: raw *Fangfeng*, FC: stir-fried *Fangfeng*, FT: charred *Fangfeng*) at various concentrations, and the y-axis represents the climbing index (0 to 1). (A) Climbing index of female *Drosophila melanogaster*. (B) Climbing index of male *Drosophila melanogaster*. Yellow bars represent FS-treated groups, blue bars represent FC-treated groups, and orange bars represent FT-treated groups. Columns show mean values ± SEM (n = 4), with individual data points representing four independent experiments. Statistical significance compared to the control group is indicated as *P < 0.05, **P < 0.01, and *P < 0.001.

Fig 5 illustrates that different processing methods of *Fangfeng*(raw *Fangfeng*, stir-fried *Fangfeng*, charred *Fangfeng*) significantly enhance the climbing ability of *Drosophila melanogaster*. All treatment groups displayed a marked improvement in climbing ability compared to the control group (K), indicating a positive impact of these methods on motor function. Notably, the FT method showed the most pronounced improvement in climbing ability across all dosages. Both female and male flies in the FT treatment groups, especially at higher dosages, achieved the highest climbing indices, outperforming the FS and FC groups. Moreover, the climbing ability of *Drosophila* demonstrated a dose-dependent enhancement with increasing doses of Radix Saposhnikoviae. For both female and male flies, higher dosages (0.04 g/mL and 0.08 g/mL) in the FT treatment groups significantly improved climbing performance compared to the lower dosage (0.02 g/mL).

The results indicate that Radix Saposhnikoviae, especially FT, exhibits significant anti-aging potential. It can effectively delay the decline in motor function in *Drosophila melanogaster*, potentially extending their lifespan and improving their physiological state.

### Effects of different preparations of *Fangfeng* on the body weight of naturally aging *Drosophila* models

In addition to lifespan and reproductive performance, body weight serves as an important physiological indicator of aging and metabolic health. To evaluate the potential impact of *Fangfeng* preparations on overall vitality, we monitored changes in body weight in naturally aging Drosophila. In the control group, the average body weight of 15-day-old flies was 6.20 ± 0.54 mg per 10 females and 3.73 ± 0.17 mg per 10 males, based on three independent replicates. These values are consistent with established physiological benchmarks for healthy *Drosophila melanogaster* [37], validating the suitability of the model for assessing treatment effects.

**Fig 6:**
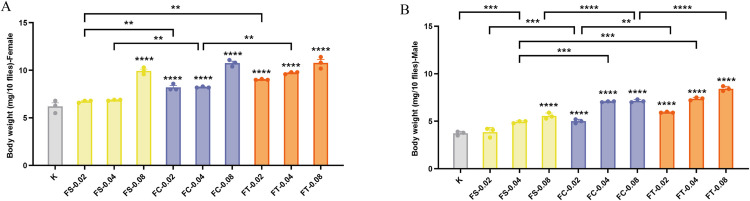
Body weight increase of aging *Drosophila melanogaster* under different *Fangfeng* treatments at 0.02, 0.04, and 0.08 g/mL dosages. The x-axis represents different *Fangfeng* treatment groups (K: control, FS: raw *Fangfeng*, FC: stir-fried *Fangfeng*, FT: charred *Fangfeng*) at various concentrations, and the y-axis represents body weight (mg/10 flies). (A) Body weight of female *Drosophila melanogaster*. (B) Body weight of male *Drosophila melanogaster*. Yellow bars represent FS-treated groups, blue bars represent FC-treated groups, and orange bars represent FT-treated groups. Columns show mean values ± SEM (n = 3), with individual data points representing three independent experiments. Statistical significance compared to the control group is indicated as *P < 0.05, **P < 0.01, ***P < 0.001, and **P < 0.0001.

Fig 6 illustrates the effects of different preparations of *Fangfeng* on the body weight of naturally aging *Drosophila melanogaster*, separated by gender. Different preparations of *Fangfeng* (raw *Fangfeng*, stir-fried *Fangfeng*, charred *Fangfeng*) significantly increased the body weight of naturally aging *Drosophila melanogaster*. Figs 6A and 6B show that the body weight of all treatment groups was significantly higher than that of the control group (K), indicating a positive impact of these preparations on the body weight of *Drosophila*. Among the various preparations, the FT method exhibited the most pronounced effect on increasing body weight across all dosages.

At a dosage of 0.08 g/mL, both female and male flies in the FT treatment groups achieved the highest body weights, surpassing those of the FS and FC groups. Specifically, compared to the control group, the average body weight of females increased from 6.20 mg/10 flies to 10.80 mg/10 flies (a 74.2% increase), and males increased from 3.73 mg/10 flies to 8.43 mg/10 flies (a 126% increase). Additionally, the body weight of *Drosophila* demonstrated a dose-dependent increase with higher dosages of Radix Saposhnikoviae. As the dosage increased from 0.02 g/mL to 0.08 g/mL, the body weight of all treatment groups significantly increased, with the most substantial gains observed at the 0.08 g/mL dosage. These findings highlight the effectiveness of Radix Saposhnikoviae, particularly the FT preparation, in enhancing the body weight of aging *Drosophila*, thereby suggesting potential benefits for improving physiological health during aging.

### Study on the anti-aging effects of aqueous extracts from different processed products of *Fangfeng* based on NMR metabolomics

The ^1^H-NMR spectra obtained from the tissues of female fruit flies in the five groups (K-3d, K-30d, FS-0.08, FC-0.08, FT-0.08) were analyzed using TopSpin 4.1.3 software. By referencing the chemical shifts and splitting patterns of the spectral peaks against the Human Metabolome Database (HMDB), the Biological Magnetic Resonance Data Bank (BMRB), and relevant literature, we identified and distinguished 27 metabolites in the NMR spectra. The results are summarized in Table S1.

**Fig 7:**
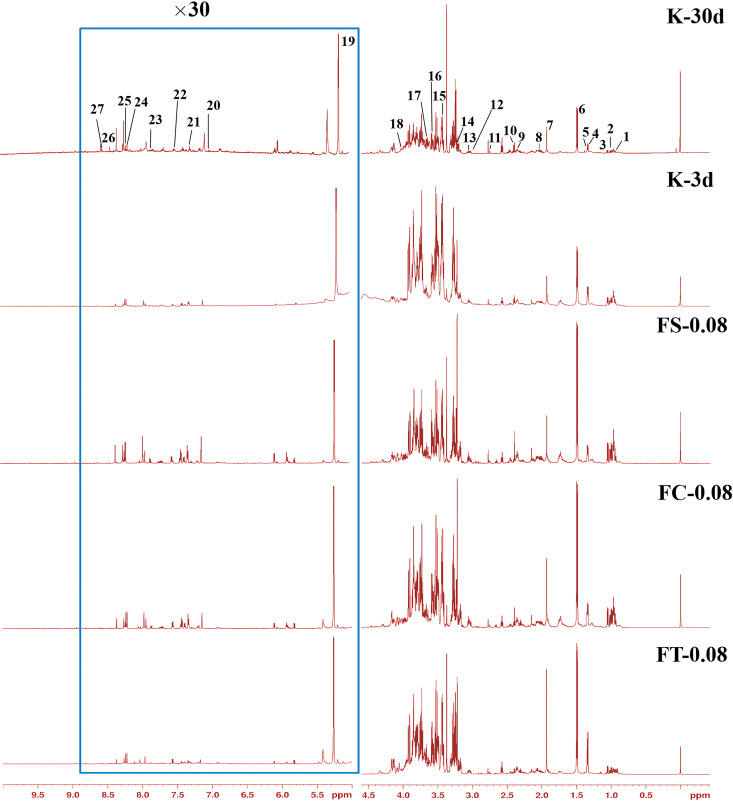
¹H-NMR spectra of female *Drosophila melanogaster* from different experimental groups. The x-axis represents the chemical shift (ppm) and the y-axis indicates signal intensity. Spectra are shown for the K-30d group (control flies aged 30 days), K-3d group (control flies aged 3 days), FS-0.08 group (flies treated with 0.08 g/mL of raw *Fangfeng*), FC-0.08 group (flies treated with 0.08 g/mL of stir-fried *Fangfeng*), and FT-0.08 group (flies treated with 0.08 g/mL of charred *Fangfeng*). Key metabolite peaks are labeled with numbers corresponding to specific compounds identified in the study. The region highlighted in blue (9.5–5.5 ppm) is magnified 30 times to show low-intensity peaks more clearly.

The ^1^H-NMR spectra of different treatment groups shown in Fig 7 (see also full original spectra in S2) reveal significant differences, indicating that the various processed forms of *Fangfeng* have markedly distinct impacts on the metabolism of fruit flies. Each treatment group exhibits unique metabolic characteristics. These differences may be attributed to the chemical composition and biological activity of each processed product.

Additionally, the ^1^H-NMR spectra of the K-30d and K-3d groups display distinct metabolic profiles, demonstrating that age significantly influences *Drosophila* metabolism. There are notable differences in the composition and concentration of metabolites between the older and younger groups, underscoring the important role of age in metabolic regulation.

The detailed list of metabolites and their chemical shifts provided in Table S1 offers a comprehensive overview of the metabolic composition of each group. This information aids in identifying and quantifying the metabolites across different groups and facilitates further analysis of the metabolic pathways and biochemical reactions. These findings provide crucial scientific insights into the metabolic regulation mechanisms of various processed forms of *Fangfeng* and their anti-aging effects.

In conclusion, multivariate statistical analysis of the ^1^H-NMR spectra enables us to uncover the significant impacts of different processing methods and ages on the metabolism of fruit flies. These discoveries not only enhance our understanding of the bioactivity and metabolic characteristics of *Fangfeng* but also offer new perspectives and theoretical support for its application in anti-aging drug development.

### ^1^H-NMR multivariate statistical analysis

**Fig 8:**
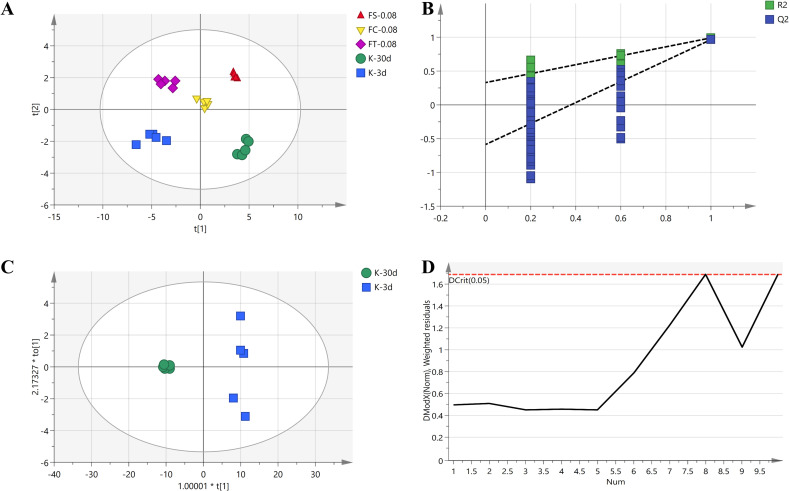
Multivariate statistical analysis of metabolites in female fruit flies from K-30d Group, K-3d Group, FS-0.08 Group, FC-0.08 Group, and FT-0.08 Group. (A: PLS-DA B: Permutation C: OPLS-DA D: DModX).

The metabolite data collected from the ^1^H-NMR spectra of female fruit flies in the K-30d, K-3d, FS-0.08, FC-0.08, and FT-0.08 groups were subjected to multivariate statistical analysis, as shown in Fig 8. Using partial least squares discriminant analysis (PLS-DA) to analyze the metabolic profiles of the five groups of fruit flies (with R^2^X=0.973, R^2^Y=0.983, Q^2^ = 0.963), the results are presented in Fig 8.

The analysis reveals significant metabolic differences between the various sample groups. Fig 8 -A (PLS-DA plot) illustrates the distribution of each sample group in multivariate space, where the distinct separation of sample points for each group (K-30d, K-3d, FS-0.08, FC-0.08, FT-0.08) indicates notable differences in their metabolic characteristics. This clear clustering phenomenon demonstrates that different treatment conditions result in markedly distinct metabolic profiles in the fruit flies, thereby validating the differential impact of each treatment on the flies’ metabolism.

Fig 8B (Permutation Test Plot) displays the R^2^ and Q^2^ values. The R^2^ values (green) and Q2 values (blue) are both high, indicating that the model has strong explanatory power and predictive capability without signs of overfitting. The permutation test shows that the actual model’s Q2 values are significantly higher than those of the random models, demonstrating the model’s high stability and reliability.

Multivariate statistical analysis of ^1^H-NMR spectra demonstrates significant differences in the metabolic profiles of fruit flies at different ages. Fig 8 -C (OPLS-DA plot) clearly illustrates the separation between the K-30d and K-3d groups, indicating distinct metabolic characteristics between the two age groups. The distinct clustering of sample points further corroborates the substantial impact of age on the metabolic pathways in fruit flies.

Fig 8 -D (DModX plot) illustrates the outlier situation in the model. The majority of sample points fall below the critical value (Dcrit), indicating the absence of significant outliers in the data.

Overall, these charts, through comprehensive multivariate statistical analysis, reveal the significant impact of different processing treatments and age on the metabolic profiles of fruit flies. These findings provide essential scientific evidence for elucidating the metabolic regulatory mechanisms and anti-aging effects of various processed forms of *Fangfeng*.

### Dynamic study of metabolites during the aging process in female *Drosophila*

**Fig 9:**
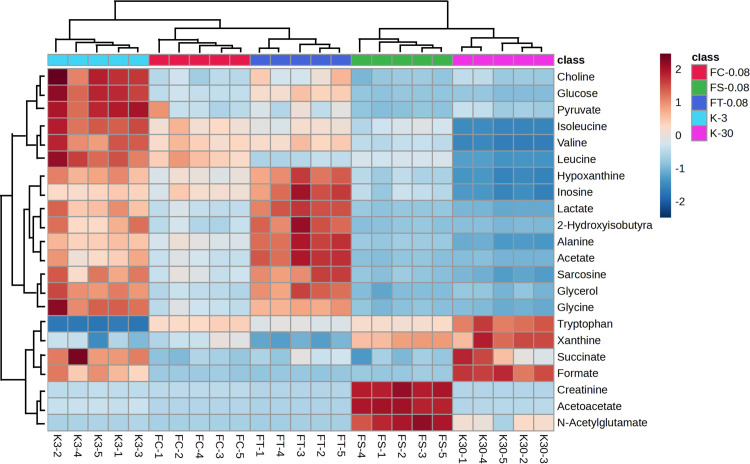
Heatmap of endogenous metabolites in female *Drosophila.* The heatmap displays the relative abundance of 24 metabolites across five experimental groups: FC-0.08 (green), FS-0.08 (blue), FT-0.08 (red), K-3 (cyan), and K-30 (pink). Data were clustered using hierarchical clustering with Euclidean distance and complete linkage. Color intensity represents metabolite levels (z-score normalization), with red indicating higher abundance and blue indicating lower abundance. Metabolites are listed on the right, and individual samples are shown at the bottom.

As shown in Fig 9 In the heatmap, colors closer to dark red indicate upregulated metabolite expression levels, while colors closer to dark blue indicate downregulated levels. The figure demonstrates the significant impact of age on the metabolic characteristics of fruit flies. In Fig 9, the heatmap shows a marked difference in color distribution between the K-30d group and the K-3d group. The K-30d group (older flies) exhibits dark red and deep blue areas, while the K-3d group (younger flies) displays a different color pattern. Significant differences are observed in the expression levels of multiple metabolites between the K-30d and K-3d groups. For example, metabolites such as glucose, lactate, and pyruvate show noticeable differences in expression levels, reflecting variations in metabolic pathways between older and younger flies. The clustering analysis in the heatmap shows that sample points from the K-30d and K-3d groups form distinct clusters, further supporting the significant differences in their metabolic profiles. This clustering phenomenon indicates that fruit flies of different ages exhibit distinct changes in their metabolic networks, reflecting differences in their physiological states.

The figure illustrates that different processing methods of *Fangfeng* significantly impact the metabolic characteristics of fruit flies. The clustering analysis in the heatmap reveals that the blank control groups (K-30d, K-3d) and the processed *Fangfeng* groups (FS-0.08, FC-0.08, FT-0.08) primarily cluster into two distinct categories. Specifically, the K-30d group clusters with the FS-0.08 group, while the K-3d group clusters with the FC-0.08 and FT-0.08 groups. This clustering pattern indicates that the different processing methods of *Fangfeng* have distinct effects on the metabolic profiles of fruit flies, resulting in two separate metabolic categories among the treatment and control groups.

Significant differences in metabolite expression levels are observed across the different processing groups. The heatmap shows that the metabolite levels in the FC-0.08 and FT-0.08 groups transition from deep blue to dark red, indicating a substantial upregulation of metabolite expression in these groups. In contrast, the FS-0.08 group exhibits less pronounced changes in metabolite expression, suggesting a weaker metabolic impact. The pronounced differences between the K-30d group (older flies) and the K-3d group (younger flies) provide a baseline for evaluating the effects of the different processed *Fangfeng* treatments. By comparing the processed *Fangfeng* groups with these control groups, the specific impacts of different treatments on metabolic profiles can be more clearly discerned.

In conclusion, the clustering analysis and the differences in metabolite expression levels in the heatmap demonstrate the significant impact of different processing methods of *Fangfeng* on the metabolic characteristics of fruit flies. These findings suggest that the chemical composition and biological activity of the various processed forms of *Fangfeng* differ, leading to distinct metabolic responses.

### Analysis of differential metabolites

**Fig 10:**
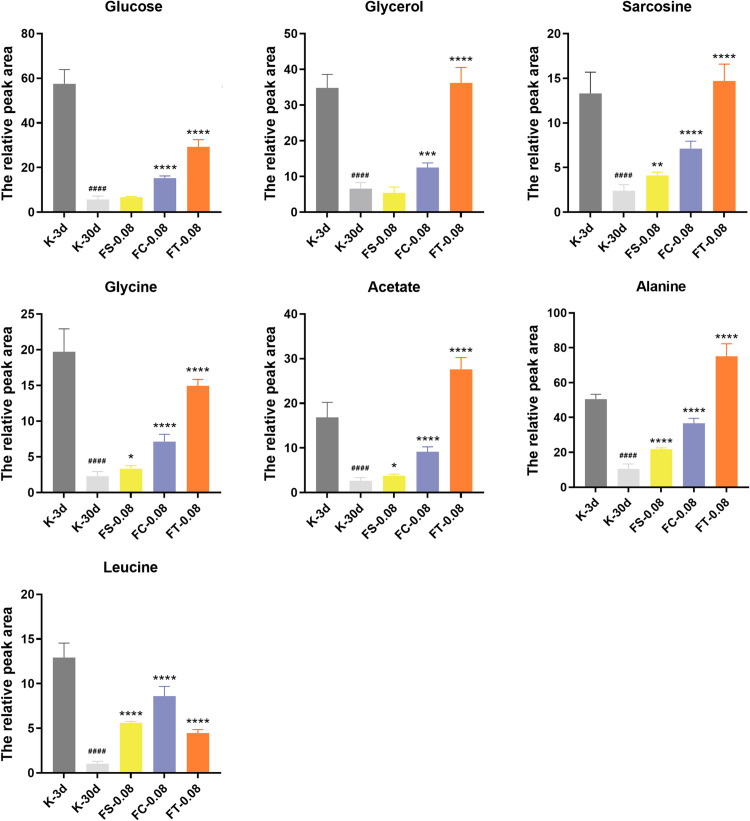
Relative peak area of differential metabolites in female *Drosophila melanogaster* treated with different *Fangfeng* preparations at 0.08 g/mL. Each panel represents one of the six significantly altered metabolites (Glucose, Glycerol, Sarcosine, Glycine, Acetate, Alanine, and Leucine). Groups include K-3d (gray), K-30d (light gray), FS-0.08 (yellow), FC-0.08 (blue), and FT-0.08 (orange). Data are presented as mean ± SEM (n = 3). Statistical significance was determined using one-way ANOVA followed by Tukey’s post hoc test: ####P < 0.0001 vs. K-3d, *P < 0.05, **P < 0.01, ***P < 0.001, **P < 0.0001 vs. K-30d.

As illustrated in Fig 10, we employed orthogonal partial least squares discriminant analysis (OPLS-DA) to analyze the metabolomic data of female fruit flies from the 30-day blank control group (K-30d) and the 3-day blank control group (K-3d) to identify differential metabolites. Metabolites were selected based on a variable importance in projection (VIP) score greater than 1, followed by independent sample T-tests (P < 0.05). Seven differential metabolites were identified: glucose, alanine, glycerol, glycine, acetate, sarcosine, and leucine. These metabolites were subjected to further statistical analysis.

The figure displays the relative peak areas of these seven differential metabolites across different treatment groups, including glucose, glycerol, sarcosine, glycine, acetate, alanine, and leucine. The metabolite levels are presented for the following 5 groups: the 3-day blank control group (K-3d), the 30-day blank control group (K-30d), the raw *Fangfeng* group (FS-0.08), the stir-fried *Fangfeng* group (FC-0.08), and the charcoal-processed *Fangfeng* group (FT-0.08).

The figure clearly indicates that age has a significant impact on the metabolic characteristics of fruit flies. By comparing the metabolite levels between the K-3d group (young flies) and the K-30d group (old flies), it is evident that all seven metabolites (glucose, glycerol, sarcosine, glycine, acetate, alanine, and leucine) show significantly lower levels in the K-30d group. The relative peak areas of these metabolites are markedly reduced in the K-30d group compared to the K-3d group, indicating that as the fruit flies age, their metabolic profiles undergo significant changes, with a general decline in metabolite levels.

These differences are statistically significant (P < 0.0001), suggesting that these changes are not random but are closely related to aging. In the figure, the symbol ### denotes the significant reduction in the K-30d group compared to the K-3d group. All seven metabolites exhibit the same trend, with levels in the K-30d group being significantly lower than those in the K-3d group. This consistency further supports the significant impact of age on metabolic characteristics.

In summary, as fruit flies age, the levels of metabolites in their bodies significantly decrease, indicating that age has a substantial effect on the metabolic characteristics of fruit flies. These data convincingly demonstrate that the metabolic activity of older flies is significantly reduced, resulting in lower levels of metabolites.

### Carbohydrate metabolism

Glucose is a primary source of cellular energy metabolism. High levels of glucose metabolism indicate adequate energy supply within cells, which is crucial for maintaining normal physiological functions and activities. Among the experimental groups, the K-3d group exhibited the highest glucose metabolism levels, while the K-30d group showed a significant reduction in glucose metabolism. This indicates that the glucose metabolism in young fruit flies is significantly higher than in older fruit flies, suggesting that the restoration of glucose metabolism levels can be considered an indicator of delayed aging in anti-aging research. Compared to the K-30d group, the glucose levels in the FC-0.08 and FT-0.08 groups significantly increased, demonstrating that these processed forms of *Fangfeng* can effectively restore glucose metabolism. The impact of different processed forms of *Fangfeng* on glucose metabolism reflects their differences in metabolic regulatory mechanisms. For instance, stir-fried *Fangfeng* (FC-0.08) and charcoal-processed *Fangfeng* (FT-0.08) significantly enhanced glucose metabolism levels, indicating that these treatments positively influence glucose metabolic pathways. In contrast, raw *Fangfeng* (FS-0.08) showed a relatively weaker effect. This variation highlights the importance of processing methods in enhancing the efficacy of *Fangfeng* in metabolic regulation and underscores the potential of specific processing techniques to improve the bioactivity of traditional medicinal herbs.

### Lipid metabolism

Glycerol, a crucial product of lipid catabolism, plays a significant role in energy metabolism and biosynthesis. As shown in the figure, there are significant differences in glycerol metabolism levels among the experimental groups. The K-3d group (young flies) exhibited the highest glycerol metabolism levels, while the K-30d group (old flies) showed a marked reduction, indicating a significant decline in glycerol metabolism with age in female fruit flies.

In the treatment groups, both stir-fried *Fangfeng* (FC-0.08) and charcoal-processed *Fangfeng* (FT-0.08) significantly increased glycerol metabolism levels compared to the K-30d group. These treatment groups demonstrated a substantial recovery in glycerol metabolism, with levels approaching or even surpassing those of the K-3d group. This suggests that different processing methods of *Fangfeng* have distinct effects on glycerol metabolism, reflecting their variations in metabolic regulatory mechanisms. Acetate is a precursor of acetyl-CoA, a core molecule in the tricarboxylic acid (TCA) cycle, which generates ATP and provides energy for cellular processes. Acetyl-CoA is also crucial for fatty acid and cholesterol synthesis. High acetate metabolism levels indicate sufficient acetyl-CoA availability for these biosynthetic pathways. The figure shows that the acetate metabolism level in the K-3d group (young flies) is significantly higher than in the K-30d group (old flies) (####), suggesting a decline in acetate metabolism with age. This decline reflects reduced energy metabolism and fatty acid synthesis capacity in aged fruit flies.

In the treatment groups: FS-0.08 group: Acetate metabolism levels showed a slight increase (*) compared to the K-30d group, indicating that raw *Fangfeng* has a limited effect on restoring acetate metabolism in aged flies. FC-0.08 group: Acetate metabolism levels significantly increased (****) compared to the K-30d group, approaching the levels seen in the K-3d group. This suggests that stir-fried *Fangfeng* effectively restores acetate metabolism in aged flies. FT-0.08 group: Acetate metabolism levels were the highest (****), even surpassing those in the K-3d group. This indicates that charcoal-processed *Fangfeng* has the most substantial effect on enhancing acetate metabolism in aged flies. Both glycerol and acetate play important roles in energy metabolism. Glycerol can be converted into glucose through gluconeogenesis, providing energy to cells. Acetate, through acetyl-CoA, enters the TCA cycle to generate ATP. Both metabolites are involved in lipid metabolism-glycerol is a product of fat breakdown and can be re-synthesized into fats, while acetate is a precursor for fatty acid synthesis via acetyl-CoA.

The metabolic levels of glycerol and acetate can serve as indicators of the metabolic state of the organism. High levels of both metabolites typically reflect active metabolic processes and adequate energy supply.

### Protein metabolism

Among the differential metabolites, 4 amino acids-sarcosine, glycine, alanine, and leucine-are related to protein metabolism. These amino acids are components of proteins, and their metabolic levels can reflect the activity of protein synthesis and degradation. As shown in the figure, the amino acid metabolism levels in the K-3d group (young flies) are generally higher than those in the K-30d group (old flies). This indicates a significant decline in protein metabolism activity with age in fruit flies, possibly due to a decrease in protein synthesis capacity or an increase in protein degradation. This age-related metabolic change reflects the degradation of metabolic function during aging.

In the different treatment groups: FS-0.08 Group: The metabolic levels of the four amino acids showed a certain degree of increase, but the overall effect was not as significant as in the other treatment groups. This indicates that raw *Fangfeng* has a limited effect on restoring amino acid metabolism in aged fruit flies. Although there was some metabolic improvement, it did not significantly reverse the age-related decline. FC-0.08 Group: The metabolic levels of sarcosine, glycine, alanine, and leucine were significantly higher than those in the K-30d group, approaching or even surpassing the levels in the K-3d group. Stir-fried *Fangfeng* demonstrated a strong effect in restoring amino acid metabolism in aged fruit flies. This significant metabolic improvement may be due to the production of certain active components during the stir-frying process, which enhance metabolic regulatory capacity and improve protein synthesis and degradation efficiency. FT-0.08 Group: The effects on the metabolic levels of the four amino acids were more complex. Under the influence of FT-0.08, the levels of sarcosine and alanine exceeded those in the K-3d group, indicating a strong anti-aging capability. The improvement in glycine metabolism with FT-0.08 was greater than that with FC-0.08 but lower than in the K-3d group. Interestingly, the enhancement effect of FT-0.08 on leucine metabolism was not as pronounced as its effects on the other three amino acids and was even weaker than that of FC-0.08.

Overall, the amino acid metabolism levels in the K-3d group (young flies) were significantly higher than those in the K-30d group (old flies), reflecting a marked decline in protein metabolism activity with age. The FS-0.08 group showed limited improvement in amino acid metabolism, failing to significantly reverse the age-related decline. In contrast, the FC-0.08 and FT-0.08 groups exhibited significant effects in restoring and enhancing amino acid metabolism in aged fruit flies. Stir-fried *Fangfeng* was particularly effective in enhancing the metabolism of sarcosine, glycine, alanine, and leucine, while charcoal-processed *Fangfeng* had a strong effect in restoring the metabolism of sarcosine and alanine.

### Metabolic pathway analysis

**Fig 11:**
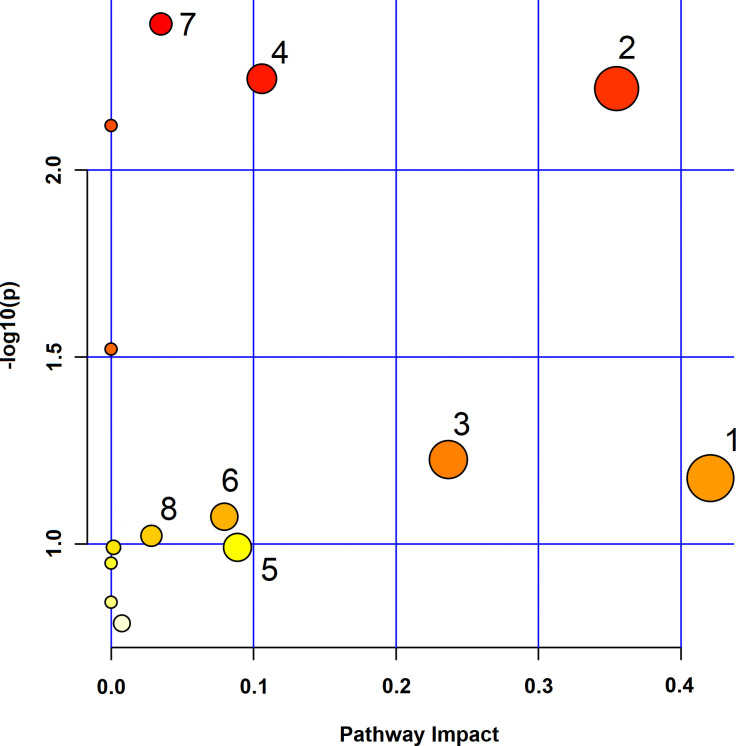
Metabolic pathway analysis by MetPA. The x-axis represents the pathway impact, and the y-axis represents the -log10(p) value. The size of each node indicates the pathway impact, and the color gradient from yellow to red indicates the significance level, with red representing the most significant pathways. Pathways are labeled numerically (1 to 8), and these labels are referenced in the manuscript text and figure legends. 1: Starch and Sucrose Metabolism; 2: Glycine, Serine, and Threonine Metabolism; 3: Glycerolipid Metabolism; 4: Glyoxylate and dicarboxylate metabolism; 5: Glutathione metabolism; 6: Pyruvate metabolism; 7: Galactose metabolism; 8: Glycolysis/ Gluconeogenesis.

The differential metabolites in female fruit flies were visualized using the MetPA platform, resulting in the identification of eight potential metabolic pathways primarily associated with carbohydrate metabolism, lipid metabolism, and amino acid metabolism. As shown in Fig 11, these pathways include: (1) starch and sucrose metabolism; (2) glycine, serine, and threonine metabolism; (3) glycerolipid metabolism; (4) glyoxylate and dicarboxylate metabolism; (5) glutathione metabolism; (6) pyruvate metabolism; (7) galactose metabolism; and (8) glycolysis/gluconeogenesis.

In this study, a comprehensive metabolomic analysis was conducted to identify key metabolic pathways significantly impacted in the context of aging, with Fangfeng used as a model. The results revealed several metabolic pathways closely associated with aging-related processes, including oxidative stress, inflammation, and energy metabolism. These pathways play critical roles in maintaining cellular homeostasis and are often disrupted during the aging process, leading to the development of age-related diseases.

Pathway 1 was identified as having the highest impact and moderate significance. This pathway likely represents a core energy metabolism route, such as glycolysis or the tricarboxylic acid (TCA) cycle. The integrity of energy metabolism is crucial for sustaining cellular functions, and disruptions in this pathway have been linked to various age-related declines, including reduced ATP production and increased susceptibility to oxidative damage. The significant impact of this pathway underscores its central role in maintaining cellular energy homeostasis, suggesting that modulating this pathway could be a potential strategy for mitigating aging effects.

Pathway 2 exhibited a high level of significance and moderate impact, indicating its critical role in the aging process. This pathway is likely associated with oxidative stress response mechanisms, which are known to be pivotal in aging. The accumulation of reactive oxygen species (ROS) and the resultant oxidative damage are major contributors to cellular aging. The involvement of this pathway suggests that *Fangfeng* may exert its anti-aging effects by enhancing cellular antioxidant capacity, thereby reducing oxidative stress and protecting cells from age-related damage.

Pathway 3, with moderate impact and significance, is likely related to lipid metabolism or protein homeostasis. These processes are essential for maintaining the structural integrity of cellular membranes and proteins, which deteriorate during aging. Disruptions in lipid metabolism can lead to membrane dysfunction, while impaired protein homeostasis is linked to the accumulation of damaged or misfolded proteins, a hallmark of aging and neurodegenerative diseases. The modulation of this pathway could therefore contribute to the preservation of cellular function in aging tissues.

Pathway 4 showed high significance but a lower impact, suggesting involvement in specific metabolic reactions crucial under certain physiological conditions, such as inflammatory responses or immune function. Chronic low-grade inflammation, often referred to as “inflammaging,” is characteristic of aging and is implicated in the pathogenesis of numerous age-related diseases. The high significance of this pathway indicates its potential involvement in modulating inflammatory responses, making it a target for anti-aging interventions.

Pathways 5 through 8 were found to have lower significance and impact but are still relevant to the aging process. These pathways might involve minor but essential metabolic adjustments, such as the synthesis or degradation of specific metabolites that contribute to overall cellular homeostasis. Even though their individual impact is lower, the cumulative effect of modulating these pathways could play a supportive role in enhancing the resilience of cells against aging-related stressors.

Overall, this study reveals the complex interplay of metabolic processes involved in aging, with *Fangfeng* significantly modulating key pathways such as energy metabolism, oxidative stress response, and inflammation. Unlike previous studies, which have largely focused on isolated pathways or effects of specific compounds, our metabolomic approach provides a more integrated and systemic understanding of *Fangfeng*’s multifaceted anti-aging mechanisms. This comprehensive analysis highlights the herb’s ability to enhance cellular resilience, offering protective effects that span multiple biochemical processes critical for aging.

Notably, the modulation of both energy metabolism and oxidative stress pathways stands out as a unique finding. While other studies have demonstrated antioxidant effects in traditional medicinal herbs, few have provided such a detailed and interconnected view of how energy homeostasis and inflammation are concurrently influenced. This integrated metabolic insight suggests that *Fangfeng* acts through multiple, converging pathways to delay aging, which contrasts with earlier studies focused on singular outcomes like glucose regulation or ROS reduction.

Our findings provide a solid foundation for future research into herb-based interventions targeting aging, setting our study apart from more narrowly focused investigations. As the first to leverage NMR metabolomics in examining *Fangfeng*’s anti-aging effects, this research opens new avenues for exploring how traditional herbs can be systematically integrated into modern therapeutic strategies for aging and age-related diseases.

### Study on the antioxidant capacity of *Fangfeng* and its processed products

In the metabolomic study, Pathway 2 demonstrated significant relevance and moderate impact, highlighting its essential role in the aging process. This pathway is likely linked to oxidative stress response mechanisms, which are critical in cellular aging. The accumulation of reactive oxygen species (ROS) and the resulting oxidative damage are well-established contributors to age-related cellular deterioration. The engagement of this pathway suggests that *Fangfeng* exerts its anti-aging effects by enhancing cellular antioxidant defenses, thereby mitigating oxidative stress and protecting cells from damage associated with aging.

**Fig 12:**
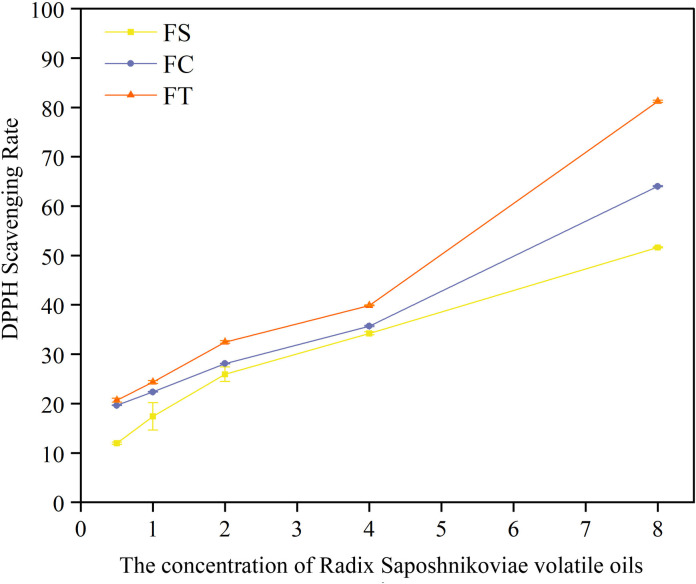
DPPH Radical scavenging activity of volatile oils from different preparations of Radix Saposhnikoviae.

Fig 12 demonstrates the DPPH radical scavenging activity of volatile oils extracted from raw *Fangfeng*(FS), roasted *Fangfeng*(FC), and carbonized *Fangfeng*(FT) at various concentrations (µL/mL). The antioxidant activities of volatile oils extracted from *Fangfeng* vary significantly depending on the processing method. The data presented in Fig 12 demonstrate that different processing methods-raw *Fangfeng*(FS), roasted *Fangfeng*(FC), and carbonized *Fangfeng*(FT)-exhibit markedly different capacities to scavenge DPPH free radicals.

Among the three preparations, the carbonized *Fangfeng*(FT) displayed the highest antioxidant activity at all tested concentrations, followed by the roasted *Fangfeng*(FC), with the raw *Fangfeng*(FS) showing the lowest activity. This is evident as the red line (FT) in the figure consistently indicates higher DPPH scavenging rates compared to the other two preparations. At the highest concentration of 8 µL/mL, the FT preparation achieved a DPPH scavenging rate of nearly 70%, significantly outperforming both FC and FS.

Furthermore, the antioxidant activity of all three preparations increased with rising concentrations, indicating a dose-dependent relationship. The DPPH scavenging rates for FS, FC, and FT all showed substantial increases as the concentration rose, especially at higher concentrations (above 4 µL/mL). This trend underscores the positive correlation between concentration and antioxidant activity. The carbonized *Fangfeng*(FT) exhibited the most potent antioxidant activity among the tested preparations, demonstrating superior efficacy in scavenging DPPH free radicals across all concentrations.

**Fig 13:**
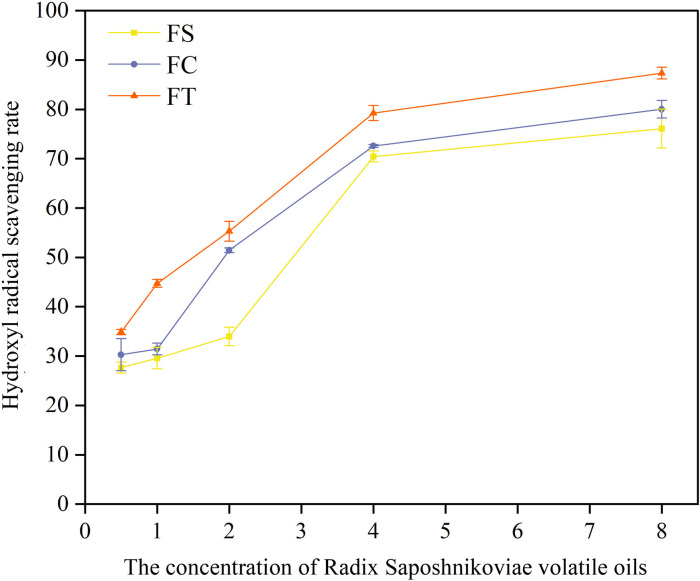
Hydroxyl radical scavenging activity of volatile oils from different preparations of Radix Saposhnikoviae.

Hydroxyl radicals are among the most reactive free radicals in the body, capable of causing severe damage to biomolecules, including DNA, proteins, and lipids. Assessing the ability of antioxidants to scavenge hydroxyl radicals can better simulate their protective effects in biological systems. To comprehensively evaluate the antioxidant capacity of *Fangfeng* and its processed products, we measured the hydroxyl radical scavenging activity of volatile oils extracted from raw *Fangfeng* and its processed products. Fig 13 illustrates the hydroxyl radical scavenging activity of volatile oils extracted from raw *Fangfeng*(FS) and its processed products: roasted *Fangfeng*(FC) and carbonized *Fangfeng*(FT) at various concentrations (µL/mL).

The antioxidant activities of volatile oils extracted from *Fangfeng*vary significantly depending on the processing method. The data presented in the figure show that different preparations-raw *Fangfeng*(FS), roasted *Fangfeng*(FC), and carbonized *Fangfeng*(FT) exhibit markedly different capacities to scavenge hydroxyl radicals.

Among the three preparations, carbonized *Fangfeng*(FT) displayed the highest antioxidant activity across all tested concentrations, followed by roasted *Fangfeng*(FC), with raw *Fangfeng*(FS) showing the lowest activity. This is evidenced by the red line (FT) in the figure, which consistently indicates higher hydroxyl radical scavenging rates compared to the other two preparations. At the highest concentration of 8 µL/mL, FT achieved a hydroxyl radical scavenging rate close to 90%, significantly outperforming both FC and FS. The antioxidant activity of all three preparations increased with rising concentrations, indicating a dose-dependent relationship. The scavenging rates for FS, FC, and FT all showed substantial increases as the concentration increased, particularly at higher concentrations (above 4 µL/mL). This trend underscores the positive correlation between concentration and antioxidant activity.

In conclusion, the dual assessment of antioxidant activities using DPPH and hydroxyl radical scavenging assays underscores the importance of processing methods in optimizing the health benefits of Radix Saposhnikoviae. The antioxidant capacity may be one of the mechanisms underlying the anti-aging effects of *Fangfeng* and its preparations.

While this study provides compelling evidence for the anti-aging effects of Radix Saposhnikoviae in Drosophila and offers metabolomic insights into its mechanisms, several limitations should be acknowledged. First, we did not assess inflammatory cytokines, which are known to be critical contributors to aging. Investigating how *Fangfeng* influences pro-inflammatory and anti-inflammatory cytokine levels would provide deeper understanding of its immunomodulatory and anti-aging potential. Second, the possible role of *Fangfeng* in age-related neurodegenerative processes was not explored in this study. Future investigations could incorporate neurobiological assessments such as neuronal apoptosis detection to evaluate its neuroprotective effects. Finally, although mTOR signaling was highlighted in the Introduction, we did not measure the expression of mTOR, FOXO, SOD, or other aging-related genes. Future research using qPCR and protein-level analyses will be essential to validate the regulatory effects of *Fangfeng* at the molecular level. These directions will help clarify the broader biological impact and translational potential of Radix Saposhnikoviae in aging intervention strategies.

## Conclusion

This study provides significant insights into the anti-aging and antioxidant effects of *Fangfeng* through an in-depth metabolomic analysis using *Drosophila melanogaster* as a model organism. The results demonstrated that charred *Fangfeng* extended the mean lifespan of *Drosophila melanogaster* from 24d to 37d, a result comparable to the lifespan extension observed with red ginseng treatment. These findings indicate that *Fangfeng* may have a similarly strong potential for promoting longevity. The metabolomic approach enabled the identification of key metabolic pathways, such as energy metabolism, oxidative stress response, lipid metabolism, protein homeostasis, and inflammatory responses, that are critically involved in the aging process and significantly modulated by *Fangfeng*. The study highlighted how the herb’s impact on these pathways contributes to its potent antioxidant properties, enhancing cellular resilience against oxidative stress-a major factor in aging. Following the discovery of oxidative stress as one of the herb’s key anti-aging mechanisms, in vitro antioxidant experiments were conducted to validate these metabolomic findings, confirming *Fangfeng*’s protective role against oxidative damage. These metabolomic findings not only underline the herb’s capacity to maintain metabolic homeostasis and delay aging but also provide a mechanistic understanding that supports its traditional use in promoting longevity. Compared to previous studies, this research provides a comprehensive metabolomic characterization of *Fangfeng*’s anti-aging effects, highlighting the significance of processing methods in enhancing its therapeutic efficacy and traditional applications. These findings may serve as a scientific reference for the integration of traditional herbal strategies in modern aging-related healthcare.

## Supporting information

S1 Table Attribution of ¹H-NMR metabolites in female *Drosophila* tissues. A table listing the proton NMR chemical shifts and metabolite assignments detected in the tissues of female *Drosophila*.
(DOCX)

S2 Fig Representative ¹H-NMR spectra of selected samples. NMR spectral profiles of samples K30, K3, FS-008, FC-008, and FT-008.
(PDF)
