# Supplementary material for: Unveiling the anti-aging of radix saposhnikoviae: A metabolomic study in Drosophila
Source: PLoS One. 2025 Aug 20;20(8):e0330274. doi: 10.1371/journal.pone.0330274 (PMC12367190; doi:10.1371/journal.pone.0330274)
Supplement: S1 Table — A table listing the proton NMR chemical shifts and metabolite assignments detected in the tissues of female Drosophila. (DOCX) [file pone.0330274.s001.docx]

**Table S1 Attribution of ^1^H-NMR metabolites in female *Drosophila* tissues**

| No. | Compound | Chemical Shift |
| --- | --- | --- |
| 1 | Leucine | 0.96（d） |
| 2 | Isoleucine | 1.01（d） |
| 3 | Valine | 0.99（d），1.04（d） |
| 4 | Lactate | 1.33（d） |
| 5 | 2-Hydroxyisobutyrate | 1.34（s） |
| 6 | Alanine | 1.49（d） |
| 7 | Acetate | 1.92（s） |
| 8 | N-Acetylglutamate | 2.02（s） |
| 9 | Pyruvate | 2.35（s） |
| 10 | Succinate | 2.41（s） |
| 11 | Sarcosine | 2.74（s），3.61（s） |
| 12 | Cysteine | 3.03（m） |
| 13 | Creatinine | 3.05（s），4.03（s） |
| 14 | Choline | 3.19（s） |
| 15 | Acetoacetate | 2.27（s），3.42（s） |
| 16 | Glycine | 3.57（s） |
| 17 | Glycerol | 3.66（dd） |
| 18 | Hippurate | 3.98（d） |
| 19 | Glucose | 5.26（d），3.91（dd）  3.83（m），3.72(m)，3.43（m） |
| 20 | Histidine | 7.08（s），7.81（s） |
| 21 | Phenylalanine | 7.34（m），7.44（m） |
| 22 | Tryptophan | 7.73（d），7.56（d） |
| 23 | Xanthine | 7.87（s） |
| 24 | Hypoxanthine | 8.22（s） |
| 25 | Inosine | 8.35（s），8.24（s），6.11（d） |
| 26 | Formate | 8.46（s） |
| 27 | IMP | 8.57（s） |
