# Supplementary material for: Unveiling the anti-aging of radix saposhnikoviae: A metabolomic study in Drosophila
Source: PLoS One. 2025 Aug 20;20(8):e0330274. doi: 10.1371/journal.pone.0330274 (PMC12367190; doi:10.1371/journal.pone.0330274)

S2_NMR_Spectra

1. K30 NMR_Spectra

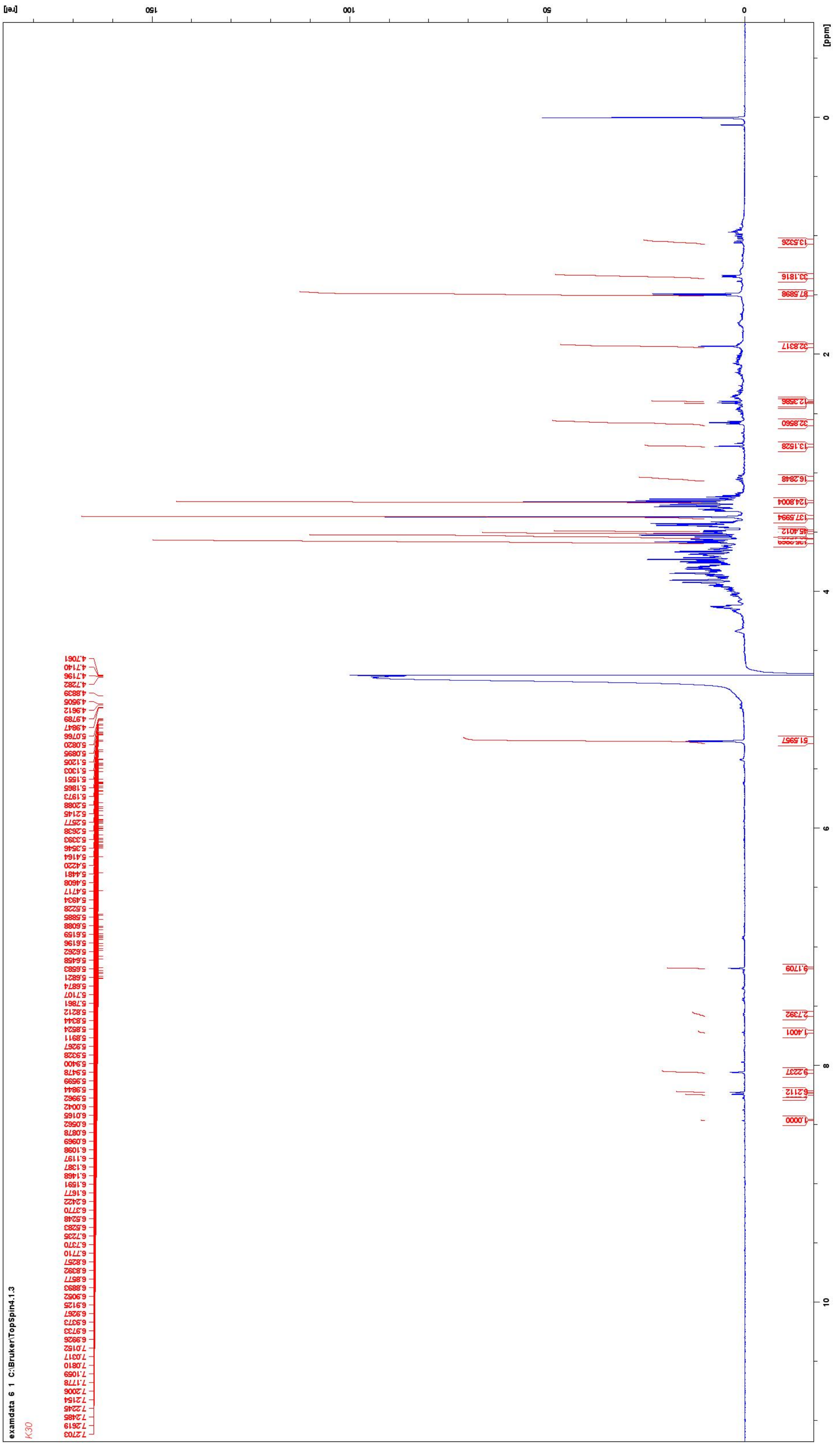

2. K3 NMR_Spectra

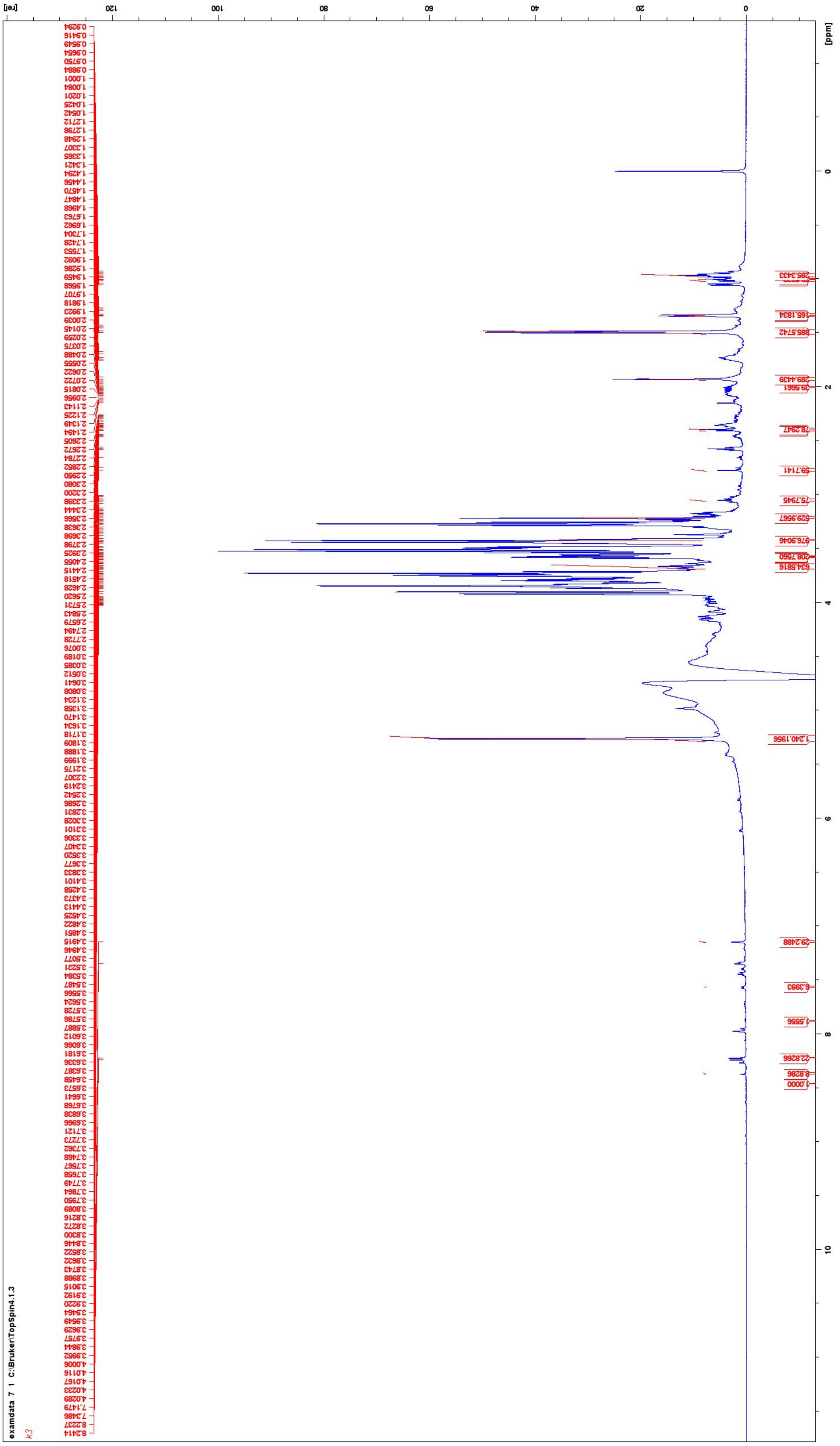

3. FS-008 NMR_Spectra

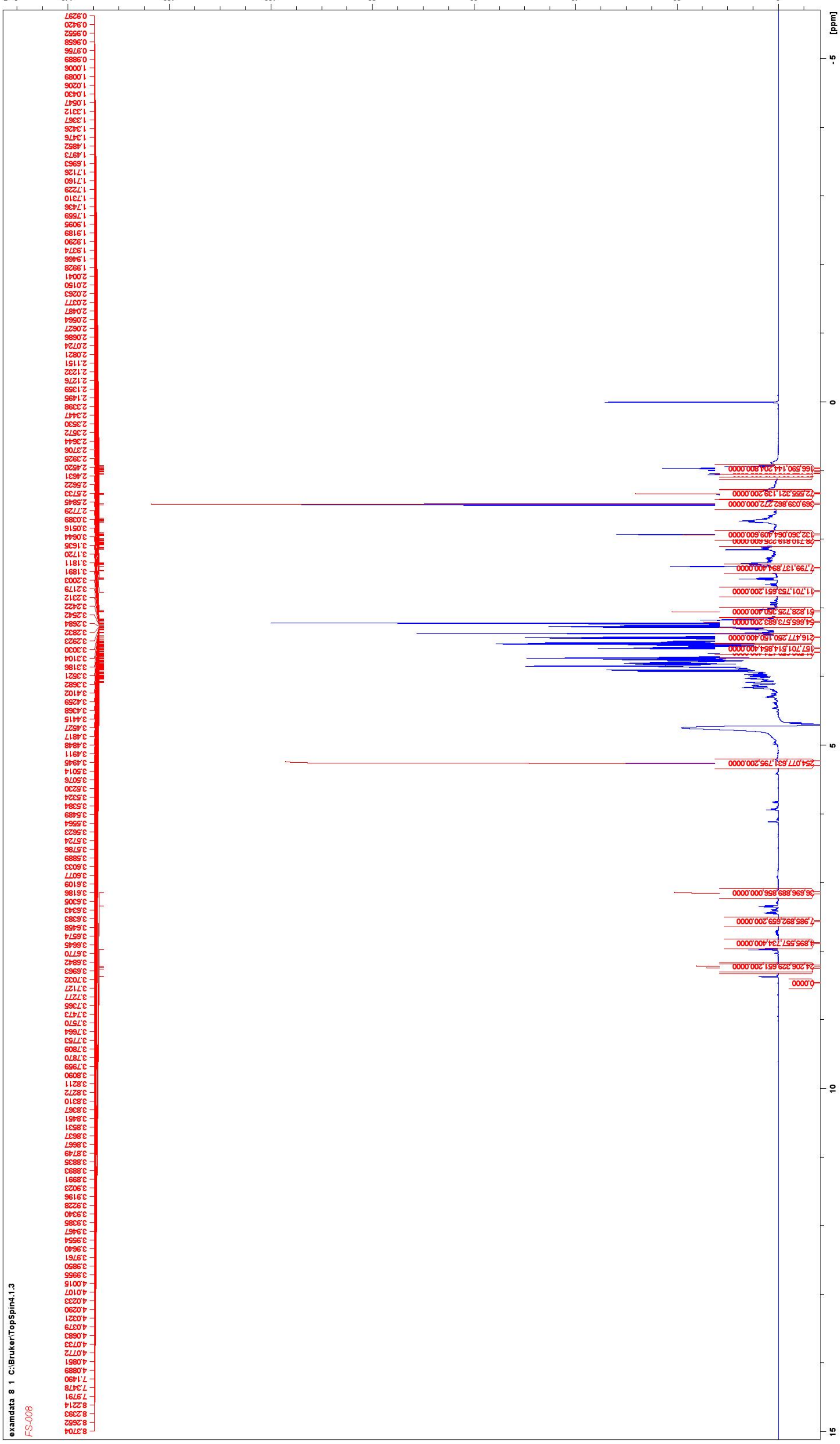

4. FC-008 NMR_Spectra

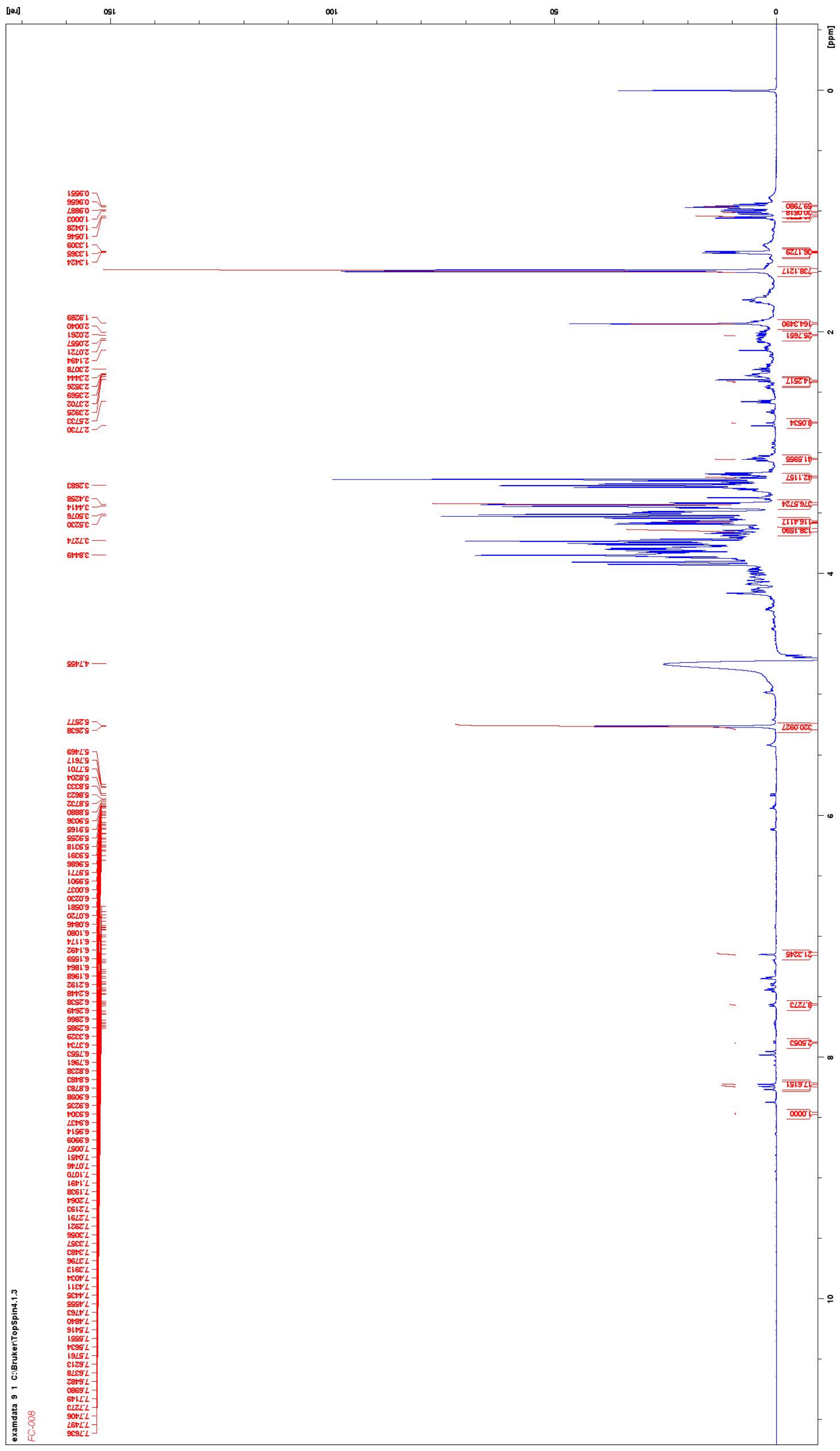

5. FT-008 NMR_Spectra

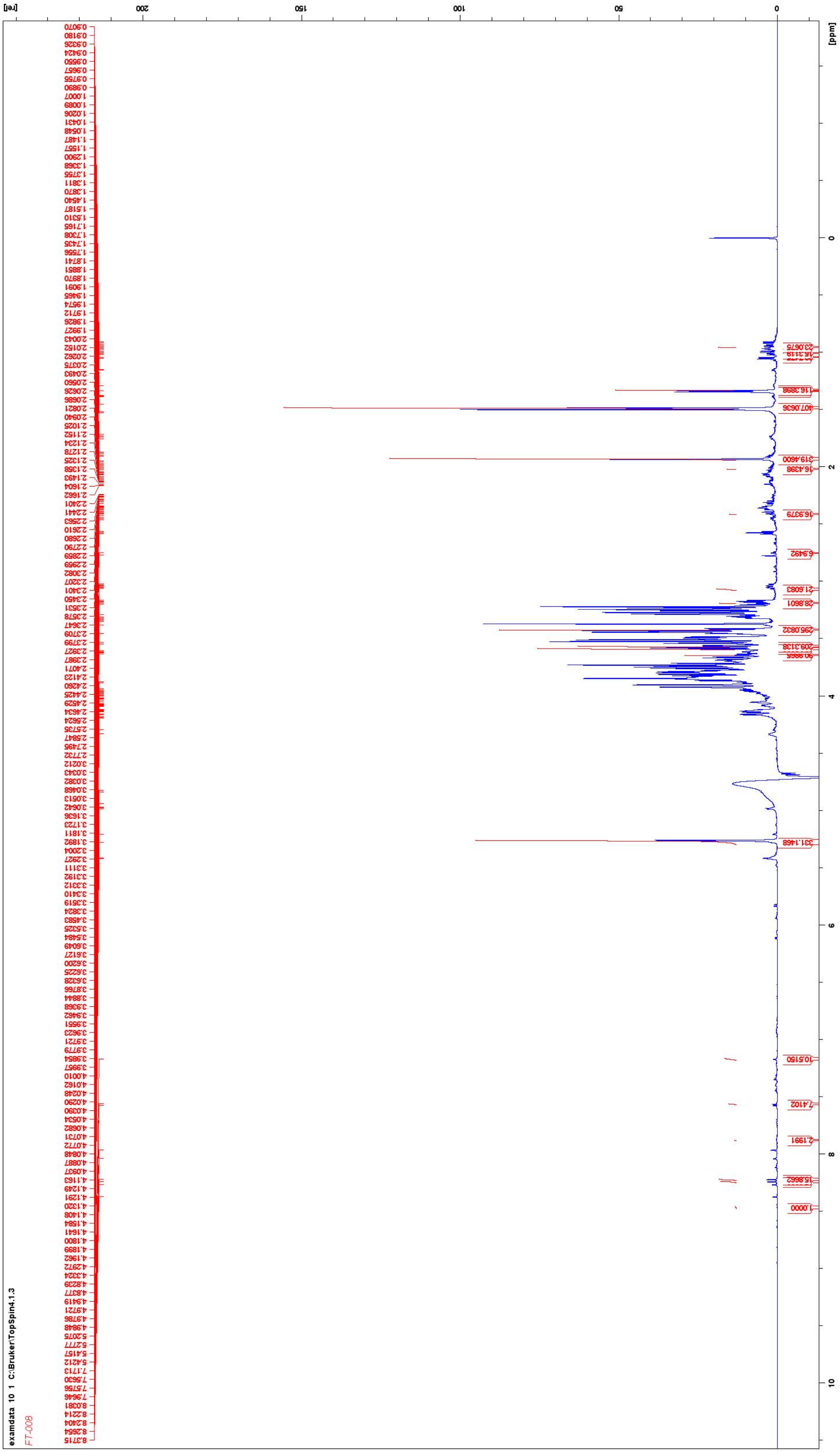

Supplement: S2 Fig — NMR spectral profiles of samples K30, K3, FS-008, FC-008, and FT-008. (PDF) [file pone.0330274.s002.pdf]
